# Dissecting the rust resistance in salt tolerant wheat germplasm

**DOI:** 10.3389/fmicb.2024.1448429

**Published:** 2024-10-24

**Authors:** Charu Lata, Pramod Prasad, Om P. Gangwar, Jayanth Kallugudi, Sneha Adhikari, Anshul Sharma Manjul, Subodh Kumar, Arvind Kumar, Neeraj Kulshreshtha, Anil Khippal, Ratan Tiwari

**Affiliations:** ^1^ICAR-IIWBR, Shimla, India; ^2^ICAR-Central Soil Salinity Research Institute, Karnal, India; ^3^ICAR-Indian Institute of Wheat and Barley Research, Karnal, India

**Keywords:** *Triticum aestivum*, *Puccinia*, seedling resistance, salt tolerance, molecular markers

## Abstract

Wheat is one of the most important food crop cultivated across the globe which ensures sustainability and food security to massive world’s population, but its production is threatened by both biotic factors like rust (caused by *Puccinia* species) and abiotic stresses such as salinity. In this study, 41 salt-tolerant wheat lines were screened for rust resistance at both seedling and adult plant stages. Rust resistance genes were characterized through gene matching technique and molecular markers analysis. *Yr2* was confirmed in 23 lines, while *Yr9* along with *Lr26*/*Sr31* were postulated in six lines with the help of SRT and molecular markers. Except for KRL2013, none showed complete resistance to all *Puccinia striiformis* f. sp. *tritici* (*Pst*). *Lr24/Sr24* genes were confirmed in HD2851 and KRL2029, and *Lr13* was detected in a maximum of 24 wheat lines, with varying reaction responses to different leaf rust pathotypes. Several lines carried additional resistance genes such as *Sr11*, *Sr28*, and *Lr68*. *Lr68* an effective race non-specific APR gene identified in 15 wheat lines with the help of *CsGs-STS* marker. Although many salt-tolerant wheat lines were susceptible to yellow rust during the seedling stage, a few lines showed APR in the years during 2020 and 2021. Three lines (KRL213, KRL219 and KRL238) showed complete resistance at adult plant stage to leaf rust. These findings offer insights into the genetic basis of rust resistance in salt-tolerant wheat, aiding breeding strategies.

## Introduction

Wheat (*Triticum aestivum* L.) is a vital food crop, contributing ~785 million tons annually from a vast cultivation area spanning 219 million hectares. It stands as a cornerstone in global agriculture, supplying the majority of calories essential for people worldwide (FAO, 2023). Wheat comes up substantially to the daily nutrition and food security for ever-growing world’s population (Rahmatov et al., 2019). World’s population depends on wheat for 20% of calories and 55% of carbohydrates in diet (Lata et al., 2022b). Approximately, 36 % of the people depend on wheat as a staple food across the globe. However, production of wheat was continuously increased due to efforts of wheat researchers and farmers, a significant research gap exists in addressing the dual challenges posed by biotic and abiotic stresses to sustain wheat productivity. The ever-growing population of world and changing climate always put a challenge for wheat producers by ever increasing demand and consumption. The increasing population is expected to hike the demand of wheat from 621 million ton (during 2004–2006) to more than 900 million ton by 2050 (Hellin et al., 2012). Several biotic and abiotic stresses pose adverse effect on wheat production throughout world. Production and productivity of wheat crop faces formidable challenges due to the prevalence of biotic stresses, such as rust diseases, and the increasing burden of abiotic stresses, notably salinity. Rust diseases are serious global threat to wheat production. Rusts caused by three species of *Puccinia*: stripe rust by *Puccinia striiformis* f. sp. *tritici* Eriks. (*Pst*); stem rust by *Puccinia graminis* f. sp. *tritici* Eriks. and E. Henn (*Pgt*) and leaf rust by *Puccinia triticina* Eriks. (*Pt*) are major biotic stress, reducing global wheat production (Prasad et al., 2022). Under favorable environmental conditions, leaf rust can cause up to 50% loss, where as stem rust and stripe rust can result in 100% loss of wheat yield. Stripe rust of wheat is a threat in 10 million hectares of Northern India, and stem rust threatens about 7 million hectares of Central and Peninsular India while leaf rust damage wherever wheat is grown in India (Bhardwaj et al., 2019). Wheat rust pathogens exhibit remarkable adaptability, often render resistant cultivars susceptible within a few cropping cycles (Hovmøller et al., 2016). Occurrence of new virulences leads to the discontinuance of imperative wheat cultivars due to rapid evolution of rust pathotypes posing a serious concern for wheat breeding. While rust resistance is crucial, an equally pressing concern is the impact of soil salinity on wheat production, particularly in salt-affected regions where wheat productivity is compromised. Within recent couple of years, several wheat rust outbreaks have been faced owing to newly evolved virulent rust pathogens. This novel virulence attacked many commercial high yielding wheat cultivars led to their complete withdrawal from wheat growing belts of India and globe.

Salinity and rust diseases are serious concerns for wheat production across the world. Nearly one billion hectares of land, approximately 25% of the global land area, is directly affected by salinity. This area is escalating up to 10 million hectares of land per year due to unsuitable irrigation practices or natural salinization (Sheoran et al., 2021; Sheoran et al., 2022). Soil salinity is a serious constraint for wheat production in various parts of the world and it has caused yield losses up to 60% leading to food insecurity. Salinity tolerance is an extremely complex quantitative trait concerning plant-specific physiological, morphological and metabolic processes. Concurrently, salinity stress, driven by soil salinization, continues to erode agricultural productivity, with detrimental effects on crop growth and yield (Yang et al., 2008). Osmotic stress and ion toxicity are two major impacts of salinity stress. Lower osmotic potential in the plant roots results higher assimilation of Na^+^ ion and decreases the Na^+^/K^+^ ratio. Uptake and transport of other important essential ions get disturbed due to this ionic imbalance which obstructs the crucial plant functions and processes (Arif et al., 2020).Therefore, a little success has been achieved in breeding of wheat varieties for salinity stress despite of rigorous efforts. Additional detrimental effects of salinity which limit the growth and yield of wheat are, cell ultra-structure alteration, membranous structure damage, production and increase in reactive oxygen species, reduction in enzymatic activity and disturbance in photosynthesis, (Dadshani et al., 2019).

Despite the need for wheat genotypes that exhibit both rust resistance and salinity tolerance, limited research has been conducted to address these dual stresses. Wheat germplasm having genes for leaf rust and yellow rust resistance normally demonstrate low level of salinity tolerance as compared to other genotypes. On other hand, salt-tolerant wheat germplasm is generally susceptible to rust (Dr. SC Bhardwaj, personal communication). Salt-tolerance is a quantitative trait and the genes governing salt tolerance are closely linked to genes of other abiotic stress than leaf rust resistance. Mexican and Pakistani wheat genotypes which showed rust resistance, exhibit sensitivity to salinity stress as compared to rust susceptible cultivars (Ali et al., 2007). To date, few studies have focused on the combined breeding for rust resistance and salinity tolerance, representing a critical research gap that this study aims to address.

There are limited studies on wheat breeding for combined stresses of salt and rust resistance. Therefore, bottom up approach is suggested to challenge these serious problems. The resistance against wheat rusts can be categorized into two broad classes: race-specific resistance and non-race-specific resistance, as outlined by Johnson (1988). Race-specific resistance, also known as seedling resistance or all-stage resistance typically exhibits a robust to moderate immune reaction, often linked with the hypersensitive response. This immune response effectively suppresses fungal infection and sporulation across all developmental stages, but its efficacy is contingent upon the presence of a corresponding avirulence gene in a pathogen (Flor, 1942; Zhang et al., 2022). This molecular dissection not only advances our comprehension of the mutual modulation of these traits but also furnishes indispensable insights for precise breeding regimes, ultimately paving the way for the development of elite wheat cultivars resilient to the challenges posed by rust diseases and salinity stress. Characterizing rust resistance in salt-tolerant wheat germplasm involves a comprehensive investigation to unravel the intricate genetic, molecular, and physiological underpinnings that govern the coexistence of disease resistance and salinity tolerance. This endeavor necessitates meticulous phenotypic assessments, encompassing diverse rust pathogens, to delineate the scope of resistance profiles and potential correlations with salt tolerance (Bakala et al., 2021). Concurrently, cutting-edge molecular methodologies, including genome-wide association studies (GWAS) and high-throughput sequencing, are harnessed to pinpoint pivotal genetic loci and candidate genes intricately linked to rust resistance (Lata et al., 2023) within salt-tolerant wheat varieties.

So, identification of rust resistance genes in salt tolerant bread wheat genotypes could be promising for developing salt-tolerant and rust resistance wheat cultivars (Lata et al., 2022a). This research is vital for filling the gap in breeding strategies that address the combined challenge of biotic and abiotic stresses. Deployment of such germplasm could avoid the epidemics of rust in near future and perform efficiently under salt-affected agro-ecosystems for sustainable wheat production. To achieve the goal of food security in adverse biotic and abiotic threats research should be focused in this direction. This study was designed to address these challenges by dissecting rust resistance in salt-tolerant advanced wheat germplasm through seedling resistance tests, adult plant resistance, and molecular approaches. The simultaneous unraveling of mechanisms underlying rust resistance and salt tolerance in wheat germplasm holds promise for devising innovative strategies to enhance crop resilience and food security.

## Materials and methods

### Host material

*A* set of 41 wheat lines including seven released cultivars/ varieties, two registered genetic stocks, two elite lines and 30 advanced lines were used for identifying rust resistance through molecular markers, SRT and APR. This set including one salt-sensitive variety, i.e., HD2851 and other 40 salt-tolerant lines of wheat were provided by Division of Crop Improvement, ICAR-Central Soil Salinity Research Institute, Karnal, India. The positive (confirmed sources *Lr, Sr,* and *Yr* genes) and negative (LWH and A-9-30) checks provided by ICAR-Indian Institute of Wheat and Barley Research, Regional Station, Flowerdale, Shimla were used for validation. Pedigree details of wheat lines are given in Table 1.

**Table 1:** List of germplasm used for identification of rust resistance genes.

S.No.	Genotype	Pedigree	Status	Released year	Source
1	KRL283	CPAN 3004/Kharchia 65//PBW 343	Cultivar	2018	CSSRI, Karnal
2	KRL210	PBW 65/2*PASTOR	Cultivar	2010	CSSRI, Karnal
3	KRL213	CNDO/R143//ENTE/MEXI_2/3/*AEGILOPS SQUARROSA* (TAUS)/4/WEAVER/5/2*KAUZ	Cultivar	2010	CSSRI, Karnal
4	KRL19	PBW 255/KRL 1–4	Cultivar	2000	CSSRI, Karnal
5	KRL1-4	Kharchia 65/WL711	Cultivar	1990	CSSRI, Karnal
6	KRL99	KRL 3-4/CIMK 2//KRL 1–4	Registered Genetic Stock	2007	CSSRI, Karnal
7	KRL3-4	HD 1982/Kharchia 65	Registered Genetic Stock	2009	CSSRI, Karnal
8	KRL19	MYNA/VUL//PRL	Elite line	Not released	CSSRI, Karnal
9	KRL238	PRL/2*PASTOR	Elite line	Not released	CSSRI, Karnal
10	Kharchia 65	KHARCHIA LOCAL/ EG 953	Cultivar	1970	CSSRI, Karnal
11	HD2851	CPAN 3004/WR 426//HW 2007	Cultivar	2006	IARI, New Delhi
12	KRL2001	KRL 99/HD 2851	Advanced Line	Yet not released	CSSRI, Karnal
13	KRL2002	CHEN/AE.SQ//2*OPATA/3/FINSI/5/W15.92/4/PASTOR//HXL7573/2*BAU/3/WBLL1	Advanced Line	Yet not released	CSSRI, Karnal
14	KRL2003	SOKOLL/WBLL1	Advanced Line	Yet not released	CSSRI, Karnal
15	KRL2004	68.111/RGB-U//WARD/3/FGO/4/RABI/5/AE.SQUARROSA (784)/6/BECARD	Advanced Line	Yet not released	CSSRI, Karnal
16	KRL2005	SOKOLL//PBW343*2/KUKUNA/3/NAVJ07/7/CHWL86/6/FILIN/IRENA/5/CNDO/R143//ENTE/MEXI_2/3/*AEGILOPS SQUARROSA* (TAUS)/4/WEAVER	Advanced Line	Yet not released	CSSRI, Karnal
17	KRL2006	UP2338*2/SHAMA/3/MILAN/KAUZ//CHIL/CHUM18/4/UP2338*2/SHAMA*2/5/PBW343*2/KUKUNA*2//FRTL/PIFED	Advanced Line	Yet not released	CSSRI, Karnal
18	KRL2007	SOKOLL/3/PASTOR//HXL7573/2*BAU/4/SOKOLL/WBLL1	Advanced Line	Yet not released	CSSRI, Karnal
19	KRL2008	CETA/AE.SQUARROSA(435)/7/2*CHWL86/6/FILIN/IRENA/5/CNDO/R143//ENTE/MEXI_2/3/ *AEGILOPS SQUARROSA*(TAUS)/4/WEAVER	Advanced Line	Yet not released	CSSRI, Karnal
20	KRL2009	KAUZ’S′/SERI//PFAU/MILAN	Advanced Line	Yet not released	CSSRI, Karnal
21	KRL2010	CHIBIA//PRLII/CM65531/3/MISR 2*2/4/HUW234 + LR34/PRINIA//PBW343*2/KUKUNA/3/ROLF07	Advanced Line	Yet not released	CSSRI, Karnal
22	KRL2011	FRET2*2/SHAMA//PARUS/3/FRET2*2/KUKUNA*2/4/TRCH/SRTU//KACHU	Advanced Line	Yet not released	CSSRI, Karnal
23	KRL2012	WAXBI*2/COPIO	Advanced Line	Yet not released	CSSRI, Karnal
24	KRL2013	SAUAL/MUTUS*2/3/WBLL1*2/KURUKU//HEILO	Advanced Line	Yet not released	CSSRI, Karnal
25	KRL2014	WBLL1*2/TUKURU//WHEAR/3/KINGBIRD #1//INQALAB 91*2/TUKURU/4/WAXBI	Advanced Line	Yet not released	CSSRI, Karnal
26	KRL2015	W15.92/4/PASTOR//HXL7573/2*BAU/3/WBLL1/5/CHRZ//BOW/CROW/3/WBLL1/4/CROC_1/AE.SQUARROSA (213)//PGO	Advanced Line	Yet not released	CSSRI, Karnal
27	KRL2016	NADI/COPIO//NADI	Advanced Line	Yet not released	CSSRI, Karnal
28	KRL2017	KRL 342/KRL 343	Advanced Line	Yet not released	CSSRI, Karnal
29	KRL2018	MUNAL #1/FRANCOLIN #1//COPIO/3/MUNAL #1/FRANCOLIN #1	Advanced Line	Yet not released	CSSRI, Karnal
30	KRL2019	KRL351/CSW18	Advanced Line	Yet not released	CSSRI, Karnal
31	KRL2020	KRL99/NW1014//BH 1146	Advanced Line	Yet not released	CSSRI, Karnal
32	KRL2021	DH4-32/PBW 343	Advanced Line	Yet not released	CSSRI, Karnal
33	KRL2022	KRL 99/Lr28//KRL35	Advanced Line	Yet not released	CSSRI, Karnal
34	KRL2023	KRL99/BARBAT	Advanced Line	Yet not released	CSSRI, Karnal
35	KRL2024	KRS1109/HW5235	Advanced Line	Yet not released	CSSRI, Karnal
36	KRL2025	CSW18/KRL283	Advanced Line	Yet not released	CSSRI, Karnal
37	KRL2026	KRL90/UP2847	Advanced Line	Yet not released	CSSRI, Karnal
38	KRL2027	KRL90/HW5235	Advanced Line	Yet not released	CSSRI, Karnal
39	KRL2028	KRL302/KRL335	Advanced Line	Yet not released	CSSRI, Karnal
40	KRL2029	CPAN3004/KH.65//KRL3-4	Advanced Line	Yet not released	CSSRI, Karnal
41	KRL2030	KRL342/KRL304	Advanced Line	Yet not released	CSSRI, Karnal

### Multi-pathotype screening through SRT

Forty-one wheat lines were screened for rust resistance at seedling stage. The pathotypes used for screening are-T, 78S84, 110S119, 110S119, P, K, 7S0, 46S119, 111S68, 79S68, 238S119 and 110S 84 (stripe rust), 11, 12A, 13–3, 12–5, 12–7,77, 77–1, 77–2, 77–5, 77–7, 77–8, 77–9, 77–10, 104–2, 107–1, 108–1, 106-1(leaf rust) and 11, 21A-2, 34–1, 40, 40A, 40–2, 40–3, 42B, 117A-1, 117–4, 117–6, 122, 184–1 (stem rust) given in Table 2 with their avirulence/virulence structure. All the pathotypes of three rust pathogens are being maintained in the national repository at ICAR – Indian Institute of Wheat and Barley Research, Regional Station Flowerdale, Shimla.

**Table 2:** List of *Puccinia* species used in this study along with virulence/avirulence pattern.

S. No.	Designation			Avirulence/virulence formula
	New	Old	North American Equivalent*	
*P. graminis* (Black Rust)				
1	79G31	11	RRTSF	*Sr7a, 8a, 8b, 9e, 22, 23, 24, 25, 26, 27, 31, 32, 33, 35, 37, 39, 40, 43, Tmp, Tt3*/ *5, 6, 7b 9a, 9b, 9c, 9d, 9f, 9 g, 10, 11, 13, 14, 15, 16, 17, 18, 19, 20, 21, 28, 29, 30, 34, 36, 38, McN*
5	75G5	21A-2	CCTJC	*Sr5, 6, 7a, 8a, 8b, 9a, 9c, 9e, 11, 12, 21, 22, 23, 24, 25, 26, 27, 29, 31, 32, 33, 35, 37, 38, 39, 40, 43, Gt, Tmp, Tt3*/*7b, 9b, 9d, 9f, 9 g, 10, 13, 14, 15, 16, 17, 19, 28, 30, 34, 36, McN*
7	10G13	34–1	MCGGP	*Sr6, 7a, 8a, 8b, 9a, 9e, 10, 11, 13, 17, 19, 21, 22, 23, 24, 25, 26, 27, 30, 31, 32, 33, 35, 36, 37, 39, 40,43, Tmp, Tt3*/*5, 7b, 9b, 9d, 9f, 9 g, 14, 15, 16, 18, 20, 28, 29, 34, 38, McN*
	104G13	40	PHDGC	*Sr2, 7a, 8a, 8b, 9a, 9b, 9c, 10, 11, 12, 13, 14, 17, 21, 22, 23, 24, 25, 26, 27, 29, 31, 32, 33, 35, 36, 37, 38, 39, 40, 43, Tmp, Tt3/5, 6, 7b, 9d, 9e, 9f, 15, 16, 19, 28,30, 34, McN*
8	62G29	40A	PTHSC	*Sr7a, 13, 21, 22, 24, 25, 26, 27, 30, 31, 32, 33, 35, 36, 37, 38, 39, 40, 43, Tmp, Tt3*/*5, 6, 7b, 8a, 8b, 9a, 9b, 9d, 9e, 9f, 9 g, 10, 11, 14, 15, 16, 17, 18, 19, 20, 23, 28, 29, 34, McN*
10	58G13-3	40–2	PKRSC	*Sr7a, 11, 13, 14, 21, 22, 23, 24, 26, 27, 29, 30, 31, 32, 33, 35, 37, 38, 39, 40, 43, Tmp*/*5, 6, 7b, 8a, 8b, 9a, 9b, 9d, 9e, 9f, 9 g, 10, 12, 15, 16, 17, 18, 19, 20, 25, 28, 34, 36, 42, Wld-1, McN, Gt*
11	127G29	40–3	PTKSF	*Sr21, 22,24, 25, 26, 27, 31, 32, 33, 35, 36, 37, 39, 40, 42, 43, Tmp, Tt3*/*5, 6, 7a, 7b, 8a, 8b, 9a, 9b, 9d, 9e, 9f, 9 g, 10, 11, 14, 15, 16, 17, 18, 19, 20, 23, 28, 29, 30, 34, 38, 44, McN, Gt*
12	7G35	42B	HRHJC	*Sr2, 5, 8a, 8b,9a, 9c, 9e, 22, 24, 25, 26, 27, 28, 29, 30, 31, 32, 35, 36, 37, 38, 39, 40, 43, Tmp, Tt3* /*6, 7a, 7b, 9b, 9d, 9f, 10, 11, 12, 13, 14, 15, 16, 17, 19, 21, 23, 33, 34, McN*
15	38G18	117A-1	HRHSC	*Sr5, 7b, 8, 12, 13, 22, 24, 25, 26, 27, 28, 30, 31, 32, 33, 35, 36, 37/6, 7a, 9a, 9b, 9c, 9d, 9e, 9f, 10, 11, 14, 15, 16, 17, 19, 21, 23, 29, 34*
16	166G3	117–4	KMGSC	*Sr5, 6, 7a, 8a, 8b, 9c, 9f, 12, 13, 14, 17, 19, 22, 23, 24, 25, 26, 27, 28, 30, 31, 32, 33, 35, 36, 38, 39, 40, 43, Tmp/2, 7b, 9a, 9b, 9e, 9d, 9e, 9 g, 10, 11, 15, 16, 21, 29, 34, 37, McN*
19	37G19	117–6	KRCSC	*Sr5, 8a, 8b, 9b, 22, 24, 25, 26, 27, 28, 30, 31, 32, 33, 35, 36, 37, Tmp*/*2, 6, 7a, 7b, 9e, 9f, 9 g, 10, 11, 12, 13, 14, 15, 16, 17, 19, 21, 23, 29, 34, McN*
20	7G11	122	RRJQC	*Sr7a, 8a, 8b, 9e, 10, 12, 14, 15, 16, 17, 18, 19, 20, 22, 24, 25, 26, 27, 28, 30, 31, 32, 33, 35, 36, 37, 38, 39, 40, 43, Tmp, Tt3*/*5, 6, 7b, 9a, 9b, 9c, 9d, 9f, 9 g, 11, 13, 21, 23, 29, 34, McN*
22	55G1	184–1	FPHSC	*Sr5, 6, 15, 18, 21, 22, 24, 25, 26, 27, 28, 29, 30, 31, 32, 33, 35, 36, 37, 38, 39, 40, 42, 43, Tmp, Tt3*/*7a, 7b, 8a, 8b, 9a, 9b, 9c, 9d, 9e, 9f, 9 g, 10, 11, 12, 13, 14, 16, 17, 19, 20, 23, 34, McN*
*P. triticina* (Brown Rust)				
	0R8	11	BBBBB	Lr1, 2a, 2b, 2c, 3, 9, 10, 12, 13, 14a, 14b, 14ab, 15, 16, 17a, 17b, 18, 19, 21, 22a, 22b, 23, 24, 25, 26, 28, 29, 30, 32, 33, 34, 36,37, 38, 39, 40, 42, 43, 44, 45, 47, 48, 49/Lr11, 20, 27 + 31, 35
	5R13	12A	FGTTL	Lr1, Lr2a, Lr2b, Lr9, Lr10, Lr13, Lr15, Lr16, Lr17a, Lr17b, Lr19, Lr24, Lr25, Lr26, Lr27 + 31, Lr28, Lr29, Lr32, Lr36, Lr39, Lr40, Lr42, Lr43, 45, 47, Lr53, Lr57, Lr58, Lr61, Lr80 / 2c, 3, 11, 12, 14a, 14b, 14ab, Lr18, Lr20, 21, 22a, 22b, 23, 30, 33, 34, 35, 37, 38, 44, Lr46 48, 49, Lr52, Lr67
	49R37	12–3	FHTTQ	Lr1, 2a, 9, 19, 20, 23, 24, 25, 28, 29, 32, 36, 39, 42, 43, 45, 47/Lr2b, 2c, 3, 10, 11, 12, 13, 14a, 14b, 14ab, 15, 16, 17a, 17b, 18, 21, 22a, 22b, 26, 27, 30, 33, 34, 35, 37, 38, 40, 44, 48,49
	29R45	12–5	FHTKL	Lr1, 2a, 9, 10, 13, 15, 19, 24, 25, 28, 29, 32, 36, 39, 42, 43, 45, 47/Lr2b, 2c, 3, 11, 12, 14a, 14b, 14ab, 16, 17a, 17b, 18, 20, 21, 22a, 22b, 23, 26, 27 + 31, 30, 33, 34, 35, 37, 38, 40, 44, 46, 48,49
	93R45	12–7	FHTTL	*Lr1, Lr2a, Lr9, Lr13, Lr15, Lr19, Lr24, Lr25, Lr28, Lr29, Lr32, Lr36, Lr39, Lr42, Lr43, Lr45, Lr47/ Lr2b, Lr2c, Lr3, Lr10, Lr11, Lr12, Lr14a, Lr14b, Lr14ab, Lr16, Lr17a, Lr17b, Lr18, Lr20, Lr21, Lr22a, Lr22b, Lr23, Lr26, Lr27 + 31, Lr30, Lr33, Lr34, Lr35, Lr37, Lr38, Lr40, Lr44, Lr46, Lr48, Lr49*
	121R63-1	77–5	THTTM	*Lr9, Lr18, Lr19, Lr24, Lr25, Lr28, Lr29, Lr32, Lr39 Lr40, Lr45 / Lr1, Lr2a, Lr2b, Lr2c, Lr3a, Lr10, Lr11, Lr14a, Lr14b, Lr14ab, Lr15, Lr16, Lr17a, Lr20, Lr21, Lr23, Lr26, Lr27 + 31, Lr30, Lr33, Lr36, Lr38, Lr42, Lr43, Lr44, Lr48, Lr49, Lr51, Lr57, Lr67*
	45R31	77	TGTKQ	*Lr9, 10, 19, 23, 24, 25, 26, 27 + 31, 28, 29, 32, 36, 39, 42, 43, 45/Lr1, 2a, 2b, 2c, 3, 10, 11, 12, 13, 14a, 14b, 14ab, 15, 16, 17, 18, 20, 21, 22a, 22b, 30, 33, 35, 37, 38, 44, 48, 49*
	109R63	77–1	THTTB	*Lr9, 17, 17a, 17b, 19, 23, 24, 25, 27 + 31, 28, 29, 32, 36, 39, 42, 43, 45, 47/Lr1, 2a, 2b, 2c, 3, 10, 11, 12, 13, 14a, 14b, 14ab, 15, 16, 18, 20, 21, 22a, 22b, 26, 30, 33, 35, 37, 38, 44, 48, 49*
	109R31-1	77–2	TGTTL	*Lr9, 19, 24, 25, 26, 28, 29, 32, 36, 39, 42, 43, 44, 45, 47/Lr1, 2a, 2b, 2c, 3,10, 11, 12, 13, 14a, 14b, 14ab, 15, 16, 17a, 17b, 18, 20, 21, 22a, 22b, 23, 27 + 31, 30, 33, 34, 35, 37, 38, 40, 48,49*
	121R63-1	77–5	THTTM	*Lr9, 19, 24, 25, 28, 29, 32, 39, 42, 43, 45, 47/Lr1,2a, 2b, 2c, 3,10, 11, 12,13, 14a, 14b, 14ab, 15, 16, 17a, 17b, 18, 20, 21, 22a, 22b, 23, 26, 27, 30, 33, 34, 35, 36, 37, 38, 40, 44,48, 49*
	121R127	77–7	TRTTL	*Lr18, 19, 24, 25, 28, 29, 32, 39, 40, 42, 45, 47/Lr1, 2a, 2b, 2c, 3, 9, 10, 11, 12, 13, 14a, 14b, 14ab, 15, 16, 17a, 17b, 20, 21, 22a, 22b, 23, 26, 27 + 31, 30, 33, 34, 35, 36, 37, 38, 43, 44, 48, 49*
	253R31	77–8	TGTTQ	Lr9, 23, 24, 25, 26, 27 + 31, 28, 29, 32, 36, 39, 45, 47/Lr1 2a, 2b, 2c, 3a, 10, 11, 13, 14a, 14b, 14ab, 15, 16, 17, 18, 19, 20, 21, 22a, 22b, 30, 33, 35, 37, 38, 44, 48, 49
	121R60-1	77–9	MHTKL	*Lr2a, Lr2b, Lr2c, Lr9, Lr19, Lr24, Lr25, Lr28, Lr32, Lr39, Lr45, Lr 47/ Lr1, Lr3a, Lr10, Lr11, Lr14a, Lr14b, Lr14ab, Lr15, Lr16, Lr17a, Lr17b, Lr18, Lr20, Lr21, Lr23, Lr26, Lr27 + 31, Lr30, Lr33, Lr36, Lr38, Lr42, Lr44, Lr46, Lr48, Lr49, Lr51, Lr57, Lr67*
	21R55	104–2	PHTTL	*Lr9, Lr 10, Lr 13, Lr 15, Lr 19, Lr 20, Lr 24, Lr 25, Lr 28, Lr 29, Lr 32, Lr 36, Lr 39, Lr 42, Lr 43, Lr 45, Lr 47 /Lr1, Lr 2a, Lr 2b, Lr 2c, Lr 3, Lr 11, Lr 12, Lr 14a, Lr 14b, Lr 14ab, Lr 16, Lr 17a, Lr 17b, Lr 18, Lr 21, Lr 22a, Lr 22b, Lr 23, Lr 26, Lr 27 + 31, Lr 30, Lr 33, Lr 34, Lr 35, Lr 37, Lr 38, Lr 40, Lr 44, Lr 48, Lr 49, Lr51, Lr57, Lr67*
	45R35	107–1	JCGPL	*Lr1, 3, 9, 10, 19, 20, 23, 24, 25, 27 + 31, 28, 29, 30, 32, 36, 38, 39, 40, 42, 43, 45,46, 47*, *Lr48, Lr49, Lr52, Lr53, Lr58, Lr62, Lr80/ Lr2a, 2b, 2c, 11, 12, 13, 14a, 18, 15, 21, 22a, 22b, 26, 33, 34, 35, 37, 38, 40, Lr51, Lr57, Lr67*
	57R27	108–1	SGTPC	*Lr3, 9, 10, 17a, 17b, 19, 23, 24, 25, 26, 27 + 31, 29, 39, 42, 45, 47 /Lr1, 2a, 2b, 2c, 11, 12, 13, 14a, 15,16, 18, 20, 21, 22a, 22b, 33, 34, 35, 36, 37, 38, 44, 48, 49,51,57,67*
	93R47	162–1	KHTTM	*Lr1, 9, 13, 14ab, 15, 19, 21, 23, 24, 25, 27 + 31, 28, 29, 32, 36, 38, 39, 40, 42, 43, 45,46, 47 / Lr2a, 2b, 2c, 3,10, 11, 12, 14a, 14b, 16, 17a,17b, 18, 20, 22a, 22b, 26, 30, 33, 34, 35, 37, 44, 48, 49,51,57,67*
*P. striformis* (Yellow Rust)				
	T(47S103)			*Yr5, Yr9, Yr10, Yr11, Yr12, Yr13, Yr14, Yr15, Yr16, Yr24, Yr26, Yrsp., Yrso, Yrsk/ Yr1, Yr2, Yr3, Yr4, Yr6, Yr7, Yr8, Yr17, Yr18, Yr19, Yr21, Yr22, Yr23, Yr25, YrA, Yrsd*
	78S84			*Yr1, Yr4, Yr5, Yr10, Yr11, Yr12, Yr13, Yr14, Yr15, Yr 16, Yr24, Yr26, Yrsk, YrA, Yrsd/ Yr2, Yr6, Yr7, Yr8, Yr9, Yr17, Yr18, Yr19, Yr21, Yr22, Yr23, Yr25, Yr27, Yrso*
	46S119			*Yr1, Yr2, Yr5, Yr10, Yr13, Yr14, Yr15, Yr16, Yr17, Yr18, Yr24, Yr28, Yrsp., Yrso*/*Yr3, Yr4, Yr6, Yr7, Yr8, Yr9, Yr11, Yr12, Yr19, Yr21, Yr22, Yr23, Yr25, Yr29*, *Yr31, YrA, Yrsd, Yrsk*
	110S84			*Yr1*, *Yr4*, *Yr5*, *Yr10*, *Yr11, Yr13, Yr14*, *Yr15, Yr16, Yr24, Yr28, Yr29*, *Yrsp., YrA*/*Yr2*, *Yr4, Yr6*, *Yr8*, *Yr9*, *Yr7*, *Yr12, Yr17, Yr18, Yr19, Yr21, Yr22, Yr23, Yr25, Yr31, YrskYrsd, Yrso*
	110S119			*Yr1*, *Yr2*, *Yr5*, *Yr10*, *Yr13, Yr15, Yr16, Yr24, Yr28, Yrsp*/*Yr2*, *Yr3, Yr4*, *Yr6*, *Yr7*, *Yr8, Yr9*, *Yr11, Yr12, Yr14, Yr17, Yr18, Yr19, Yr21, Yr22, Yr23, Yr25, Yr29*, *Yr31, YrA, Yrsd, Yrsk, Yrso*
	P(46S103)			*Yr1, Yr5, Yr9, Yr10, Yr11, Yr12, Yr13, Yr14, Yr15, Yr16, Yr24, Yr26, Yrsp., Yrso, Yrsk/ Yr2, Yr3, Yr4, Yr6, Yr7, Yr8, Yr17, Yr18, Yr19, Yr21, Yr22, Yr23, Yr25, YrA, Yrsd*
	K(47S102)			*Yr4, Yr5, Yr9, Yr10, Yr11, Yr12, Yr13, Yr14, Yr15, Yr16, Yr24, Yr26, Yrsp., Yrso, Yrsk/ Yr1, Yr2, Yr3Yr6, Yr7, Yr8, Yr17, Yr18, Yr19, Yr21, Yr22, Yr23, Yr25, YrA, Yrsd, Yrso*
	7S0			*Yr2*, *Yr3*, *Yr4*, *Yr5*, *Yr8*, *Yr9*, *Yr10*, *Yr11, Yr12, Yr14, Yr17, Yr18, Yr19, Yr21, Yr22, Yr23, Yr25, Yr29, Yr31, YrA, Yrsd, Yrsk, Yrso*/*Yr1*, *Yr6*, *Yr7*
	238S119			*Yr1*, *Yr5*, *Yr10*, *Yr13, Yr14*, *Yr15, Yr16, Yr24, Yr28*/*Yr2*, *Yr3*, *Yr4*, *Yr6*, *Yr7*, *Yr8*, *Yr9*, *Yr9* (Riebesel47/51), *Yr11, Yr12, Yr17, Yr18, Yr19, Yr21, Yr22, Yr23, Yr25, Yr29*, *Yr31, YrA, Yrsd, Yrsk, YrSo*
	111S68			*Yr1*, *Yr4*, *Yr5*, *Yr10*, *Yr11*, *Yr12*, Yr13, *Yr14*, *Yr15*, *Yr 16*, *Yr24*, *Yr26*, *Yrsk*, *YrA/ Yr2*, *Yr6*, *Yr7*, *Yr8*, *Yr9*, *Yr17*, *Yr18*, *Yr19*, *Yr21*, *Yr22*, *Yr23*, *Yr25*, *Yr27*, *Yrso*, *Yrsd*
	79S68			*Yr4Vil*, *Yr5*, *Yr9, Yr10, Yr13, Yr15, Yr16, Yr24, Yr27, YrA*, *Yrso/ Yr1*, *Yr2* (Kalyansona), *Yr3*, *Yr4Hyb46, Yr6*, *Yr7, Yr8*, *Yr11, Yr12, Yr14, Yr17, Yr18, Yr19, Yr21, Yr22, Yr23, Yr25, Yr29, Yr31, YrA, Yrsd*

#### Inoculation and disease assessment

Four to five seeds of each line were sown in aluminum pans/trays (size: 29 × 12×7 cm) filled with a mixture of fine loam soil and farmyard manure (3:1 ratio). Each tray could accommodate one susceptible check and 18 test wheat lines. These trays were maintained in rust-free microclimate rooms at 20°C after sowing. One week-old seedlings were inoculated with fresh urediniospores suspended in a light weight, non-phytotoxic isoparaffinic oil (Soltrol, Chevron Phillips Chemical Asia Pvt. Ltd., Singapore) in a concentration of 20 mg spores per 5 mL oil using an atomizer. A full set of differentials (Bhardwaj, 2012) was also inoculated along with test lines for seedling resistance test to confirm purity of pathotypes. List of pathotypes of all three rusts used in this experiment are given in Table 2. After inoculation seedlings were sprayed with a fine mist of water and incubated for 24 h in dew chambers having high humidity and specific temperature conditions, i.e., 12 ± 2°C; 20 ± 2°C; and 22 ± 2°C for stripe rust, leaf rust, and stem rust, respectively. Subsequently, the inoculated plants were transferred to temperature controlled conditions. Infection types were scored after 14 days of inoculation using a 0–4 scale (Stakman et al., 1962). Gene matching technique was used to identify possible genes governing seedling resistance in 41 wheat varieties. Stakman et al. (1962) formula was followed for scoring of infection types (ITs) on the 0–4 scale with slight modifications as proposed by Luig (1983), in which IT0 represents the lowest incompatible resistant reaction while IT4 depicts fully compatible susceptible reaction while ITX, referred to as mesothetic and classed as resistant (produces a mixture of incompatible and compatible infection types on the same leaf). Postulation of resistance genes was performed by comparing IT patterns of the pathotype range on test lines with those of controls with known resistance genes (Nayar et al., 1997). Pathotypes that are avirulent to a known resistance gene with high infection types (ITs) on a test cultivar indicated that the cultivar did not possess the gene in question.

### Screening through molecular markers

#### DNA isolation, polymerase chain reaction and product analysis

Green leaves from 5 to 7 days old seedlings were used for genomic DNA isolation by following the method of Murray and Thompson (1980) after some basic modifications. DNA quality was checked on 1% agarose gel electrophoresis and quantified on nanodrop (DS-11 FX + Spectrophotometer/Fluorometer, Jenway). Further, polymerase chain reaction (PCR) were performed in final reaction volume 25 μL with concentrations of 50 ng genomic DNA, 12 μL of 2X PCR Master Mix, containing all the necessary ingredients [Taq polymerase, MgCl2, deoxy-ribonucleotides (dNTPs) from Thermo Fisher according to user manual, 1 μL (10 pm) of each forward and reverse primer and nuclease free water]. Identification of rust resistance genes in theses wheat varieties was performed with microsatellite markers for known rust resistance genes (Table 3). PCR were performed in Bio-metra Thermal Cycler, Analytic Jena using a touchdown profile (SSR markers)with initial de-naturation at 95°C for 10 min, followed by 35 cycles of denaturation at 94°C for 60 s, annealing at (according to primer) for 30–60 s and extension at 72°C for 60 s. PCR products were separated on 2.5% agarose gels using a Bio-Rad Sub-Cell 192. To determine the fragment size pUC19 DNA/MspI 100 bp (HpaII) was run along with samples as a ladder. Stained with ethidium bromide and visualized under UV light using the digital Gel Imaging System (Bio-Rad).Gene Mapper v 4.0 software (Applied Biosystems) was used for scoring of SSR allele. Positive and negative control DNAs were run with each primer for appropriate analysis and repeated for accuracy of the results. Fourteen previously validated molecular markers were used for identification of APR and seedling rust resistance genes (Table 3).

**Table 3:** Summary of molecular markers closely linked with, their primer sequences, expected sizes of PCR products and PCR conditions.

Sl. No.	Gene	Marker	Annealing Temp	Primer sequence	Size of amplicon	References
1	*Yr18/Lr34/Sr57*	*csLV34*	55°C	F5’GTTGGTTAAGACTGGTGATGG 3’ R5’ TGCTTGCTATTGCTGAATAGT 3’	150/229	Lagudah et al. (2006)
2	*Lr19-Sr25*	*GB*	50°C	F5’ CAT CCT TGG GGA CCT C 3’ R5’ CCA GCT CGC ATA CAT CCA 3’	130	Prins et al. (2001)
3	*Yr9/Lr26/ Sr31*	*iag95-STS* *SCSS30.2*	55°C 55°C	F5’ CTCTGTGGATAGTTACTTGATCGA 3’ R5’ CCTAGAACATGCATGGCTGTTACA 3’ F5’ GTCCGACAATACGAACGATT 3’ R5’ CCGACAATACGAACGCCTTG 3’	1,100/null	Mago et al. (2005)
4	*Lr24/Sr24*	*Sr24#50*	56°C	F5’ FCCCAGCATCGGTGAAAGAA 3’ R5’ ATGCGGAGCCTTCACATTTT 3’	200/null	Spielmeyer et al. (2003)
5	*Yr17/Lr37/Sr38*	*VENTRIUP*	65°C	F5’ AGGGGCTACTGACCAAGGCT 3’ R5’ TGCAGCTACAGCAGTATGTACACAAAA 3’	259/null	Helguera et al., 2003
6	*Yr15/Yr24*	*GWM11* *BARC 8*	56 (TD) 54°C	F5’ GGATAGTCAGACAATTCTTGT 3’ R5’ GTGAATTGTGTCTTGTATGCTTCC 3’ F5’ GCGGGAATCATGCATAGGAAAACAGAA 3’ R5’ GCGGGGGCGAAACATACACATAAAAACA 3’	215/200	Peng et al. (2000)
7	*Lr68*	*CsGs-STS*	56°C	F5’ AAGATTGTTCACAGATCCATGTCA 3’ R5’ GAGTATTCCGGCTCAAAAAGG 3’	385/null	Herrera-Foessel et al. (2012)
8	*Sr2*	*GWM533*	52°C	F 5’ AAGGCGAATCAAACGGAATA 3’ R 5’ GTTGCTTTAGGGGAAAAGCC 3’	120/variations	Spielmeyer et al. (2003)
9	*Sr28*	*wPt7004*	60°C	F5’ CTCCCACCAAAACAGCCTAC 3’ R5’ AGATGCGAATGGGCAGTTAG 3’	194/166	Rouse et al. (2012)
10	*Lr32*	*WMC43*	52°C	F5’ TAGCTCAACCACCACCCTACTG 3’ R5’ ACTTCAACATCCAAACTGACCG 3’	346	Thomas et al., 2010
11	*Yr10*	*psp3000*	55°C	F5’ GCAGACCTGTGTCATTGGTC 3’ R5’ GATATAGTGGCAGCAGGATAC 3’	260/240	Bariana et al. (2002)
12	*Yr 5*	*Yr5_insertion*	55°C	5’-CTC ACG CAT TTG ACC ATA TAC AAC T 3’ 5’- TAT TGC ATA ACA TGG CCT CCA GT-3’	1281/507	Chen et al., 2003
13	*Lr67 - Yr46 - Sr55 - Pm46 - Ltn3*	*CFD71* *Lr 67 PLUSHSUT*	55°C 58°C	5’- CAA TAA GTA GGC CGG GAC AA-3’ 5’- TGT GCC AGT TGA GTT TGC TC-3’ F5’ TTATCCACGTTAGGCTCAAGT 3’ R5’ GCATCGTTTGCTTTGATTTTTGC 3’	198	Hiebert et al. (2010)

### APR screening through poly house and field screening

Four to five seeds of every line were planted under controlled conditions (in separate screen house at 10–12°C; 20–22°C and 22 ± 2°C for stripe, leaf, and stem rusts, respectively) in a mixture of soil loam and FYM in 3:1 ratio (w/w) supplemented recommended dose of nitrogen: phosphorus: potassium per square meter, maintained optimum temperature under poly-house as per standardized procedure at ICAR-IIWBR, Shimla (Bhardwaj et al., 2010). Light intensity of about 15,000 lux for 12 h was maintained for illuminating the plants. NILs having known APR genes, *Yr*, *Lr* and *Sr* genes carrying Indian wheat material and susceptible wheat variety A-9-30 and Agra Local were also planted to compare adult plant resistance response of tested lines. Urea (20 g/square meter) was added after 20 days of sowing to promote optimal growth of the plants. Similar set of wheat lines planted under field conditions at ICAR-CSSRI, Karnal. Adult plants were inoculated with mixture of pathotypes stripe rust (46S119, 110S119, 238S119, 47S103, 110S84), leaf rust (12–5, 77–1, 77–5, 77–9, 104–2), and stem rust (11, 40-A, 117–6, 21A-2 and 122) on 43–55 growth stage (boot just visible to one half of the ear emerged) as per modified Zadok’s scale (Tottman et al., 1979). Infection types of the adult plants were recorded 14 days after the inoculation. APR responses were inferred on the basis of seedlings reactions and adult plants grown under the same set of conditions with same pathotypes. The modified Cobb scale (Peterson et al., 1948) was used to determine the percentage of severity. The experiment was repeated for the wheat lines in which APR was observed.

## Results

### Identification of seedling resistance genes

To identify rust resistance genes (*Lr*, *Sr,* and *Yr*) in 41 wheat lines, seedling resistance test (SRT) was conducted and with the help of gene matching technique for all three rust with set of pathotypes.

#### Stripe rust seedling response

Eleven *Pst* pathotypes were used to postulate *Yr* genes at seedling stage in these wheat cultivars. In seedling reaction assay, presence of *Yr2* was confirmed in 23 wheat lines based on susceptible and resistant reactions against different pathotypes. On the other hand, *Yr9* was confirmed in six wheat lines on the basis of *Lr26* gene postulations in leaf rust SRT and molecular markers and showed resistance for most of the pathotypes used in this experiment. No wheat line showed complete resistance for all *Pst* races used in this experiment except KRL2013. This line showed ITs ranges from 0 to 2^−^. List of ITs along with postulated genes for *Pst* races are presented in Table 4.

**Table 4:** Rust resistance gene postulated based on multi-pathotype data at seedling stage.

S. no.	Variety/Line	*Yr* genes	*Lr* genes	*Sr* genes	S. no.	Variety/line	*Yr* genes	*Lr* genes	*Sr* genes
1	KRL283	*Yr9+*	*Lr26 + 23 + 10*	*Sr31+*	22	KRL2011	*Yr2+*	*Lr13+*	*Sr11+*
2	KRL210	*Yr2+*	*Lr23 + 10*	*Sr28+*	23	KRL2012	*Yr2+*	*Lr13+*	*–*
3	KRL213	*Yr2+*	*Lr13+*	*Sr28+*	24	KRL2013	*Yr9+*	*Lr26 + 23 + 1+*	*Sr31+*
4	KRL19	*Yr2+*	*Lr13+*	*R*	25	KRL2014	*Yr2+*	*Lr13 + 2a + 1+*	*Sr30+*
5	KRL1-4	*Yr2+*	*Lr13+*	*Sr28+*	26	KRL2015	*Yr9+*	*Lr10 + 1+*	*-*
6	KRL99	*-*	*Lr13+*	*Sr28+*	27	KRL2016	*Yr2+*	*Lr13+*	*Sr11+*
7	KRL3-4	*-*	*Lr13 + 10 + 1+*	*Sr11+*	28	KRL2017	*Yr9+*	*Lr26 + 1+*	*Sr31+*
8	KRL119	*-*	*Lr13 + 10 + 1+*	*Sr11+*	29	KRL2018	*YrA+*	*Lr13 + 1+*	*Sr57 + 11+*
9	KRL238	*-*	*Lr13+*	*Sr5 + 11+*	30	KRL2019	*Yr2+*	*Lr13+*	*Sr11+*
10	Kharchia65	*–*	–	*–*	31	KRL2020	*Yr2+*	*Lr13+*	*Sr7b + 11+*
11	HD2851	*–*	*Lr24 + R*	*Sr24+*	32	KRL2021	*Yr2+*	*Lr13 + 10 + 1*	*–*
12	KRL2001	*–*	*Lr13 + 2a*	*Sr28+*	33	KRL2022	*Yr2+*	*Lr10 + 3 + 13+*	*Sr28+*
13	KRL2002	*Yr2+*	*Lr23 + 10*	*Sr30+*	34	KRL2023	*–*	*Lr13 + 3+*	*–*
14	KRL2003	*–*	*Lr23 + 10+*	*Sr28+*	35	KRL2024	*Yr9, Yr A+*	–	*–*
15	KRL2004	*Yr2+*	*Lr23 + 10+*	*R*	36	KRL2025	*Yr2+*	*Lr13 + 10+*	*–*
16	KRL2005	*Yr2+*	*Lr23 + 10+*	R	37	KRL2026	*Yr2+*	*Lr13 + 1+*	*Sr7b + 11+*
17	KRL2006	*–*	*Lr13 + 3+*	*Sr28*	38	KRL2027	*–*	*Lr13 + 1+*	*Sr7b + 11+*
18	KRL2007	*Yr2+*	*Lr23 + 10+*	*–*	39	KRL2028	*Yr2+*	*Lr13 + 10 + 1+*	*Sr7b + 11+*
19	KRL2008	*Yr2+*	*Lr10 + 1+*	*Sr11+*	40	KRL2029	*Yr9+*	*Lr26 + R+ Lr24+*	*Sr 31 + 24+*
20	KRL2009	*Yr2+*	*Lr23+*	*Sr28+*	41	KRL2030	*YrA+*	*Lr13 + 10+*	*–*
21	KRL2010	*Yr2+*	*Lr23 + 3+*	*Sr28+*					

#### Leaf/brown rust seedling response

Seventeen *Pt* races were used for evaluation of seedling leaf rust response. Resistant and susceptible reactions to each of the races along with the postulated genes are presented in Table 4. *Lr24/ Sr24* postulated in HD2851 and KRL2029 wheat genotypes which show resistance for all pathotypes of leaf rust and stem rust pathogens at seedling stage. Six wheat lines (KRL283, KRL2013, KRL2015, KRL2017, KRL2024 and KRL2029) showed the presence of *Lr26*. Presence of these two genes was also confirmed by molecular markers analysis. Other wheat lines showed the presence of combinations of previously described *Lr* genes or new *Lr* gene/s. *Lr13* gene present in maximum 24 wheat lines which is postulated with differential reaction responses to different pathotypes of leaf rust pathogen.

#### Stem/black rust seedling response

Seedling response of wheat genotypes to *Pgt* races and postulated genes are presented in Table 4. Majority of the wheat lines showed seedling resistance toward the *Pgt* races 21A-2, 34–1, 42B, 117A-1 and 117-6with infection types (ITs) ranging between 0 to 2. Few wheat lines showed seedling resistance toward the more virulent *Pgt* races 11, 40, 40A, 40–2, 40–3, 117–4, 122 and 184–1. The resistance gene *Sr11* was postulated in maximum 12 wheat genotypes followed by *Sr28* that was postulated in 10 wheat lines. *Sr*31 found in six wheat genotypes (KRL283, KRL2013, KRL2015, KRL2017, KRL2024 and KRL2029). HD2851 and KRL2029 showed resistance toward all tested *Pgt* races due to presence of *Sr24*. However, no gene is postulated in KRL19, KRL2004 and KRL2005 because these were resistant to tested races. Thus, these lines either carry combinations of previously described genes or new resistance gene/s.

### Molecular markers assay for rust resistance genes

**Figure 1:**
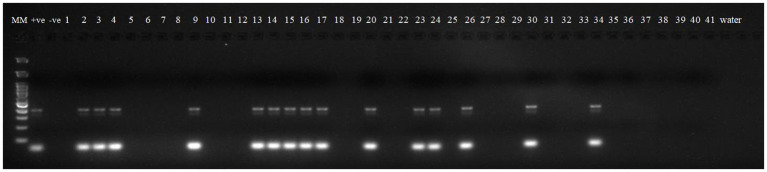
Electrophoresis on 2.5% agarose gel showing marker *Sr24#50* indicated the presence of *Lr24/Sr24* gene complex. Lane 1 (MM)- Gene Ruler 100 bp DNA ladder; Lane 2 (+ve)-*Lr24* NIL as positive check for gene *Lr24*; Lane 3 (−ve)-LWH as negative check; Lane 4–44—wheat genotypes 1 –41; Lane 45, 46 (W)- water.

**Figure 2:**
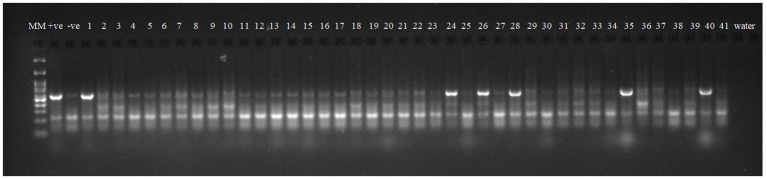
Electrophoresis on 2.5% agarose gel showing the marker *CsGs-STS* indicated the presence of the gene *Lr68.* Lane 1 (MM)-GeneRuler 100 bp DNA ladder; Lane 2 (+ve-)-*Lr68* NIL as positive check for gene *Lr68*; Lane 3 (−ve)- LWH as negative check; Lane 4–44—wheat genotypes 1–41, Lane 45, 46 (W)- water.

**Figure 3:**
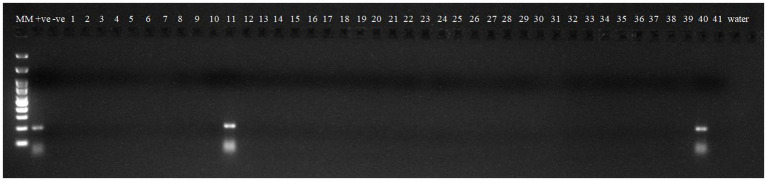
Electrophoresis on 2.5% agarose gel showing marker *SCSS30.2* indicated the presence of *Yr9/Lr26/Sr3* gene complex. Lane 1 (MM)- Gene Ruler 100 bp DNA ladder; Lane 2 (+ve)-*Yr9* NIL as positive check for gene *Yr9*; Lane 3 (−ve)-LWH as negative check; Lane 4–44 —wheat genotypes 1–41; Lane 45, 46(W)- water.

Robust molecular markers were applied on isolated DNA of wheat lines to ascertain additional rust resistance in these wheat lines. The presence of 200 bp amplicon produced by dominant marker *Sr24*#50 indicated the presence of *Lr24/Sr24* gene complex in two wheat lines, i.e., HD2851 and KRL2024 (Figure 1). These two wheat lines confirmed the presence of *Lr24/Sr24* gene by the molecular marker showed complete resistance against all the pathotypes of leaf rust and stem rust pathogen. *Lr68* (non-race specific gene) was identified through linked molecular marker *CsGs-STS*. Fifteen wheat lines (KRL210, KRL213, KRL19, KRL238, KRL2002, KRL2003, KRL2004, KRL2005, KRL2006, KRL2009, KRL2012, KRL2013, KRL2015, KRL2019, and KRL2023) confirmed the presence of *Lr68* gene with an amplification of 385 bp band size (Figure 2). *Lr19/Sr25* gene was checked with marker GB, but not a single cultivar confirmed the presence of respective 130 bp amplicon for this gene complex. *Yr9/Lr26/Sr31* was validated by SCSS30.2 marker which gives a sharp band of 550 bp in six wheat genotypes namely KRL283, KRL2013, KRL2015, KRL2017, KRL2024 and KRL2029 (Figure 3). *Yr5*_insertion, psp3000 and *GWM11/BARC8* markers were used to explore the presence of *Yr5*, *Yr10* and *Yr15* genes, respectively, in salt tolerant lines; nonetheless none of the cultivars were positive for these markers.

### Adult plant resistance for stripe rust

Evaluations of adult plant resistance for all three rusts was conducted at ICAR-IIWBR, RS, Shimla and ICAR-CSSRI, Karnal. Significant level of disease pressure was observed, with severities reaching 100S in susceptible controls. Interestingly, certain accessions that did not exhibit noticeable seedling resistance showed high levels of adult plant resistance (APR). Most salt tolerant wheat lines were found susceptible for yellow rust during seedling stage. Thus, despite susceptibility at seedling stage for 46S119, 110S119, 110S84 and 238S119, few wheat lines showed APR during 2020 and 2021. Among 41 salt tolerant wheat lines, only six (KRL213, KRL2003, KRL2007, KRL2025, KRL2026 and KRL2027) were considered as resistant (0R to TMR disease severity) at adult plant stage under field conditions (Table 5). Arrays of variation for resistance to leaf rust were observed at adult plant stage in salt tolerant wheat lines. Several lines found fully resistant with 0R score at adult plant stage. Some of these lines showed resistance in seedling stage too. However, three salt tolerant wheat lines (KRL213, KRL19 and KRL238) showed complete resistance at adult plant stage, while these were susceptible at seedling stage against tested pathotypes; 22R45 (12–5), 119R63 (77–1), 121R63-1 (77–5), 121R60-1 (52 or 77–9) and 21R55 (104–2) (Table 5). Evaluation of adult plant resistance for *Pgt* races is difficult due to resistance at seedling stage in salt tolerant wheat germplasm for tested pathotypes; 79G31(11), 62G29(40-A), 37G19 (117–6), 75G5 (21A-2) and 7G11 (122).

**Table 5:** Adult plant resistance score of salt tolerant wheat germplasm.

S. no.	Variety/Line	*P. striiformis*	*P. triticina*	*P. graminis*	S. no.	Variety/Line	*P. striiformis*	*P. triticina*	*P. graminis*
1	KRL283	0R	0	5MS	22	KRL2011	5MR	5MS	0
2	KRL210	0R	20S	20MS,S	23	KRL2012	0R	0R	0
3	KRL213	5MR	5R	10MR	24	KRL2013	TMR	0R	0
4	KRL19	40S	0R	TMR	25	KRL2014	0R	0R	5R
5	KRL1-4	60S	-	20MS	26	KRL2015	0R	5S	5MR
6	KRL99	10S	20MS	20MS,S	27	KRL2016	0R	10R	TMS
7	KRL3-4	30S	40S	60S	28	KRL2017	10MS	0R	10MR
8	KRL119	10S	40S	0	29	KRL2018	0R	0R	TR
9	KRL238	0R	0R	5MR	30	KRL2019	10S	–	5MR
10	Kharchia65	60S	40S	TMS	31	KRL2020	5MR	–	0
11	HD2851	30S	0R	5MR	32	KRL2021	–	0R	–
12	KRL2001	20S	10S	20MS	33	KRL2022	–	0R	40S
13	KRL2002	10S	10MR	20MR	34	KRL2023	10S	5S	5R
14	KRL2003	TMR	0R	10MS	35	KRL2024	0R	0R	TR
15	KRL2004	5MR	0R	20MS	36	KRL2025	5MR	20MS	TMR
16	KRL2005	0R	0R	20MS	37	KRL2026	5MR	0R	10MS
17	KRL2006	0R	5R	5S	38	KRL2027	10MR	0R	10MS
18	KRL2007	0R	10R	20S	39	KRL2028	5S	0R	5MR
19	KRL2008	TMR	0	10MR	40	KRL2029	5S	0R	10MR
20	KRL2009	5MR	–	10S	41	KRL2030	–	–	20R
21	KRL2010	5S	–	10S					

## Discussion

*Triticum aestivum* (bread wheat) is considered as the most significant grain crop among all the cereals. Considering human consumption, wheat is ranked 1st among grain-producing crops globally (Giraldo et al., 2019). In this study, the emphasis is placed on addressing both biotic and abiotic stresses. Specifically, the research aimed to identify seedling resistance genes in salt-tolerant wheat varieties by analyzing their resistance responses against different rust pathogens (stripe rust, leaf rust, and stem rust). Salt stress and rust diseases are deadly combination for wheat crop and difficult to provide tolerance to both stresses. Salt tolerant wheat germplasm are generally found susceptible for rust diseases (Lata et al., 2022a). Limited research has been conducted on rust diseases and salinity stress simultaneously, leaving this area largely unexplored and in need of further study. Some Mexican and Pakistani wheat accessions were screened for both stresses. Among the wheat accessions sourced from Mexico, a diverse spectrum of genotypes was observed, comprising five distinct groups. Remarkably, apart from the genotypes endowed with resistance against wheat leaf rust, the salinity tolerances of these Mexican genotypes exhibited minimal variations. This trait was similarly discernible among genotypes from Pakistan, possessing resistance against leaf rust and yellow rust, which displayed significantly reduced salinity tolerances (Ali et al., 2007). To date, more than 80 leaf rust resistance genes, 83 stripe rust resistance genes and 60 stem rust resistance genes are officially cataloged in wheat (Li et al., 2020; Kumar et al., 2021) and only a few genes are known to confer pleiotropic effect on the resistance for example *Lr34*/*Yr18*/*Pm38*/*Sr57* (Singh et al., 2012), *Lr46*/*Yr29*/*Pm39*/*Sr58* (Singh et al., 2013), *Lr67*/*Yr46*/*Pm46*/*Sr55* (Herrera-Foessel et al., 2011), and *Lr68* (Herrera-Foessel et al., 2012). Most of these catalogued genes showed race-specific resistance. The study employs a gene postulation technique with differential sets of wheat cultivars to determine the presence of specific rust resistance genes (*Yr*, *Lr* and *Sr*) in the wheat genotypes. In the present study the primary resistance genes identified through seedling and adult plant responses, along with molecular marker analysis, included yellow rust resistance genes; *Yr2*, *Yr9*, *YrA* and leaf rust resistance gens; *Lr1*, *Lr2a*, *Lr3*, *Lr10*, *Lr13*, *Lr23*, *Lr24* and stem rust resistance genes; *Sr5*, *Sr7b*, *Sr11*, *Sr24*, *Sr28*, *Sr30*, *Sr31*, *Sr57*. Gaining insights into resistance mechanisms and mitigating the risk of genetic erosion, which can lead to the swift decline in the efficacy of utilized genes, necessitates the thorough evaluation of novel genetic resources. This evaluation process should encompass multiple facets, encompassing the utilization of molecular markers to pinpoint resistance genes, alongside an examination of the reaction patterns exhibited by both seedlings and adult plants in commercial varieties and advanced breeding lines when confronted with different pathotypes rusts (Li et al., 2016; Gangwar et al., 2022; Lata et al., 2023). Wheat varieties grown throughout diverse regions in India during 2015 to 2019 predominantly possess rust resistance genes including *Yr2*, *Yr9*, *YrA*, *Yr18*, and *Yr27*. During the studied period resistance against rust in approximately 60% of cultivars is controlled by Gene *Yr2*, followed by *Yr9* (25%) and *YrA* (12%). Other genes have been identified in only a few cultivars. It’s important to note that none of these identified rust resistance genes are effective against all Indian *Pst* population (Gangwar et al., 2021). Resistance and susceptibility analysis of these varieties against the different races showed the presence of various rust resistance genes. In these salt tolerant genotypes 23 entries out of 41 showed presence of *Yr2* and some additional unidentified resistance followed by four entries possessing *Yr9* and three entries carry *YrA*. The severe yellow rust issue affecting India’s primary wheat varieties results from the over reliance on specific seedling resistance genes, namely *Yr2*, *Yr9*, and *Yr27*. These genes have been used without incorporating other important R genes like *Yr10*, *Yr15*, and *Yr24/Yr26* in the cultivated varieties. As a consequence, rapidly evolving and virulent races of the *Pst* pathogen have managed to overcome the resistance provided by these major single genes over time. The breakdown of this resistance has raised concerns among wheat breeders, emphasizing the urgent need to broaden the genetic diversity of future Indian wheat varieties. This can be achieved by integrating multiple yellow rust resistance genes, thus enhancing the overall resilience of the crops against the evolving and aggressive *Pst* races (Singh V. K. et al., 2015). Presently, breeders have embarked on rust resistance breeding efforts by incorporating rust-resistant sources that possess the potent genes *Yr5*, *Yr10*, and *Yr15*. Several cultivars, such as PBW752 (carrying *Yr10*) and PBW757 (carrying *Yr15*), have been developed and released for cultivation. These initiatives reflect a strategic shift toward integrating multiple effective genes to enhance resistance in wheat varieties, marking a positive step in combating the challenges posed by rust pathogens (Gupta et al., 2022).

Presence of *Lr24/Sr24* is postulated in HD2851 and KRL2029, which exhibit resistance reactions across multiple rust pathotypes. This gene provides resistance to almost all *Pgt* pathotypes found in India accept 34–1 and 40–1 were found virulent on *Sr24* (Jain et al., 2013; Prasad et al., 2023). Rest of the pathotypes were unable to establish a compatible relationship with *Sr24*-containing wheat varieties (HD2851 and KRL2029). At seedling stage, most of the salt tolerant wheat germplasm showed resistance for tested *Pgt* pathotypes due to presence of *Sr28*, *Sr11*, *Sr5*, *Sr24*, *Sr28*, *Sr30*, *Sr31*, *Sr57* and *Sr7b*. The frequency of low virulence within the Indian population of *Puccinia graminis* f. sp. *tritici* (*Pgt*) is evident on *Sr31*, *Sr24*, and *Sr30*, as elucidated by Prasad et al. (2022). These specific genes, among the ensemble of 10 *Sr* genes postulated across 41 wheat varieties emerge as potential candidates for strategic gene deployment strategies (Table 4). The study identifies several wheat genotypes with resistant genes, including *Lr24/Sr24* in HD2851 and KRL2029, *Lr26* in KRL283, KRL2013, KRL2017, and KRL2029, and *Lr13* in multiple wheat lines. The study identifies the presence of *Sr11* in 12 wheat genotypes, *Sr28* in 10 wheat genotypes, and *Sr31* in six wheat varieties (KRL2013, KRL2017, and KRL2029).Hence, these specific varieties could be strategically chosen for precise gene deployment efforts for stem rust in India. In Indian wheat varieties, commonly found *Sr* genes include *Sr5*, *Sr7a*, *Sr7b*, *Sr11*, *Sr24*, *Sr28*, *Sr30*, and *Sr31*, with *Sr57* being a crucial gene for durable resistance. Additionally, slow rusting *Sr* genes like *Sr55*, *Sr56*, *Sr57*, and *Sr58*, when combined with other potent major genes, represent a valuable and versatile resource that can be effectively utilized (Singh R. P. et al., 2015).

Resistance to wheat rusts can be broadly classified into two main types: race-specific and non-race-specific. Race-specific resistance is characterized by its specificity and short-lived nature, mainly because it operates on specific interactions between plant genes and pathogen avirulence genes. This type of resistance is quickly overcome by evolving pathogens. In contrast, non-race-specific resistance offers a more enduring defense. It involves genes with minor to intermediate effects, providing a broader spectrum of protection against various pathogen strains (Lagudah, 2011). Plants with this type of resistance are vulnerable during the seedling stage but develop resistance as they mature, a phenomenon known as slow rusting. Slow rusting is associated with adult plant resistance (APR) and offers long-term protection against rust diseases. When considered individually, the effects of these adult plant resistances (APR) genes are moderate. However, their significance becomes apparent when they interact with other major genes and various minor quantitative trait loci (QTLs). These interactions result in additive effects, leading to the development of highly durable resistance. Combining APR genes in a single cultivar can lead to nearly complete immunity or a high level of resistance. This dual classification provides valuable insights for researchers and breeders aiming to enhance wheat varieties’ resilience to rust pathogens (Wellings, 2011; Huerta-Espino et al., 2020).

Among the 41 tested wheat lines, 15 were confirmed to carry the *Lr68* gene using a linked molecular marker, CsGs-STS, which resulted in the amplification of a specific 385 bp band (Lata et al., 2022a). Under field conditions, out of the 41 salt-tolerant wheat lines studied, only six (KRL213, KRL2003, KRL2007, KRL2025, KRL2026, and KRL2027) demonstrated resistance (indicated by a severity rating of 0R to TMR disease) during the adult plant stage when exposed to 46S119, 110S119, 110S84 and 238S119 yellow rust pathotypes. It’s probable that conducting tests on potential non-specific resistance genes like *Lr34*, *Lr46*, and *Lr67* would yield limited or unproductive results (Huerta-Espino et al., 2011).

Variability in resistance to leaf rust was observed among salt-tolerant wheat lines during the adult plant stage. Some lines exhibited full resistance, scoring 0R during the adult plant stage, indicating effective resistance during adult growth. Interestingly, a subset of these lines also displayed resistance at the seedling stage. However, it is noteworthy that three specific salt-tolerant wheat lines (KRL213, KRL219, and KRL238) demonstrated complete resistance during the adult plant stage, despite being fully susceptible at the seedling stage when exposed to tested pathotypes (29R45, 109R63, 121R63-1, 121R60-1, and 21R55). Assessing adult plant resistance to specific *Pgt* races poses challenges due to the presence of resistance during the seedling stage in salt-tolerant wheat germplasm tested against pathotypes such as 79G31(11), 62G29(40-A), 37G19(117–6), 75G5(21A-2), and 7G11(122). While it is conceivable that adult plant resistance might exist in these wheat lines, accurately evaluating it remains difficult, making it uncertain to draw definitive conclusions (Prasad et al., 2023).

In conclusion, the research paper outlines the findings of a comprehensive study on the presence of rust resistance genes in salt-tolerant wheat lines. By examining seedling responses, molecular markers, and adult plant resistance, the researchers provide insights into the genetic basis of rust resistance in these wheat genotypes. These findings emphasize the complexity of rust resistance gene interactions and highlight the need for further research to fully understand and utilize these genetic traits for wheat improvement.

## Data Availability

The original contributions presented in the study are included in the article/Supplementary material, further inquiries can be directed to the corresponding author.
