# Supplementary material for: Dissecting the rust resistance in salt tolerant wheat germplasm
Source: Front Microbiol. 2024 Oct 24;15:1448429. doi: 10.3389/fmicb.2024.1448429 (PMC11540707; doi:10.3389/fmicb.2024.1448429)
Supplement: Supplementary file 1 [file Data_Sheet_1.docx]

Abbreviations- ASR- All Stage Resistance

APR- Adult Plant Resistance

NG- Not Germinated

**Table S1. APR Yellow Rust (*P. striformis*)**

| **S.N.** | **Yellow Rust (Seedling Response)** | | | | Final APR Response |
| --- | --- | --- | --- | --- | --- |
|  | **46S119** | **110S119** | **110S84** | **238S119** |  |
| 1 | 0; | 3+ | 3 | 3+ | 0R |
| 2 | 0; | 3+ | 2- | 3+ | 0R |
| 3 | 3+ | 3+ | 33+ | 3+ | 5MR |
| 4 | 3+ | 3+ | 0; | 3+ | 40S |
| 5 | 0; | 3+ | 0; | 3+ | 60S |
| 6 | 3 | 3+ | 3+ | 3+ | 10S |
| 7 | 3+ | 3+ | 3+ | 3+ | 30S |
| 8 | 2- | 3+ | 3+ | 33+ | 10S |
| 9 | 3+ | 0; | 0; | 0; | 0R |
| 10 | 3+ | 3+ | 3+ | 3+ | 60S |
| 11 | 3 | 3+ | 3+ | 3+ | 30S |
| 12 | 3+ | 3+ | 3+ | 3+ | 20S |
| 13 | 3+ | 3+ | 0; | 3+ | 10S |
| 14 | 3 | 3 | 3+ | 3+ | TMR |
| 15 | 0; | 3+ | 0; | 3+ | 5MR |
| 16 | 3+ | 3 | 0; | 3+ | 0R |
| 17 | 3+ | 2- | 3+ | 3+ | 0R |
| 18 | 3+ | 3 | 3+ | 3+ | 0R |
| 19 | 3+ | 3+ | 0; | 3+ | TMR |
| 20 | 33+ | 3+ | 0; | 3+ | 5MR |
| 21 | 3+ | 3+ | 2- | 3+ | 5S |
| 22 | 0; | 3+ | 0; | 3+ | 5MR |
| 23 | 3+ | 3+ | 0; | 3+ | 0R |
| 24 | ;- | 0; | 2- | 0; | TMR |
| 25 | 0; | 3+ | 33+ | 3+ | 0R |
| 26 | 0; | 3+ | ;1 | 3+ | 0R |
| 27 | 0; | 3+ | 33+ | 3+ | 0R |
| 28 | 0; | 0; | 3+ | 3+ | 10MS |
| 29 | 3+ | 3+ | ; | 3+ | 0R |
| 30 | 3+ | 3+ | 3+ | 3+ | 10S |
| 31 | ;- | 3 | 0; | 3+ | 5MR |
| 32 | 0; | 3 | 2 | 3+ | NG |
| 33 | 3 | 3+ | 33+ | 3+ | NG |
| 34 | 3 | 0; | 3+ | 3+ | 10S |
| 35 | 0; | 3+ | 0; | 3+ | 0R |
| 36 | 3+ | 3+ | 3+ | 3+ | 5MR |
| 37 | 3+ | 3+ | 3+ | 3+ | 5MR |
| 38 | 3+ | 3+ | 3+ | 3+ | 10MR |
| 39 | 0; | 2- | 3+ | 3+ | 5S |
| 40 | 0; | 3+ | 3+ | 3+ | 5S |
| 41 | 0; | 3+ | 0; | 3+ | - |
|  |  |  |  |  |  |

**Table S2. APR Black Rust (*P. graminis*) 79G31(11), 62G29, 37G19 (117-6)**

| S.N. | Black Rust (Seedling Response) | | | | | APR Score |
| --- | --- | --- | --- | --- | --- | --- |
|  | **79G31**  **(11)** | **62G29**  **(40-A)** | **37G19**  **(117-6)** | **75G5**  **( 21A-2)** | **7G11 (122)** |  |
| 1 | 2-- | 2-- | 0; | ; | 0; | 5MS |
| 2 | 3+ | 3 | 22+ | 22+ | 2-- | 20MS,S |
| 3 | 33+ | 23 | 2-- | 12 | 2- | 10MR |
| 4 | 2-- | 2- | 0; | 2- | 0; | TMR |
| 5 | 2-- | 0; | 2-- | 2-- | 2- | 20MS |
| 6 | 3 | 22+ | 12 | ;1 | 12 | 20MS,S |
| 7 | 3+ | 3+ | 2-- | 2-- | 33+ | 60S |
| 8 | 33+ | 2- | 2-- | ;- | 3+ | NG |
| 9 | 0; | 22+ | 0; | 0; | 0; | 5MR |
| 10 | 3+ | 3+ | 2-- | 2-- | 3+ | TMS |
| 11 | 2+ | 2- | 2-- | 2-- | ; 1N | 5MR |
| 12 | 33+ | 12 | 0; | 0; | 2+C | 20MS |
| 13 | 3+ | 2- | 2-- | 2-- | 2-- | 20MR |
| 14 | 33+ | 2- | 2-- | 12 | 22-C | 10MS |
| 15 | 2- | 2- | 2-- | 2-- | 2-C | 20MS |
| 16 | 2- | 2- | 12 | 2-- | 12 | 20MS |
| 17 | 2- | 23C | 0; | ; | 22+ | 5S |
| 18 | 12 | 2- | 12 | 2-- | 2- | 20S |
| 19 | 2+C | 2- | 2-- | 2-- | 2- | 10MR |
| 20 | 33+ | 2- | 0; | 12 | 2- | 10S |
| 21 | 33+ | 2CN | 12 | ; 1N | 2-- | 10S |
| 22 | 2C | 2- | 0; | ; | ; | NG |
| 23 | 12 | 12 | 0; | ; | ;- | NG |
| 24 | 2-- | 2- | ;1 | ; | ;1 | NG |
| 25 | 2-- | 2-- | ; | 0; | ; | 5R |
| 26 | 12 | 2- | 2-- | 0; | 0; | 5MR |
| 27 | 0; | 2- | 0; | ; | ;1 | TMS |
| 28 | 0; | 2-- | 0; | 2-- | 2- | 10MR |
| 29 | 2- | 0; | 0; | ; | ; | TR |
| 30 | 2-- | 2- | ;- | 2-- | ;1 | 5MR |
| 31 | 12 | ;1 | ;1N | ; | 3+C | NG |
| 32 | 2- | 2+ | 0; | 0; | 2- | - |
| 33 | 3+ | 2- | 0; | 0; | 22+ | 40S |
| 34 | 0; | 0; | ;- | 2 | 0; | 5R |
| 35 | 2 | 2-- | 2-- | 2 | 2-- | TR |
| 36 | 2 | 2-- | ;- | 2 | 2-- | TMR |
| 37 | 2+C | 33+ | 2-- | 2 | 3+ | 10MS |
| 38 | 22+C | 3+C | 12 | 2 | 3+ | 10MS |
| 39 | 12C | 3+ | ;1 | ; | 0; | 5MR |
| 40 | 0; | 2- | ;- | ; | 2-- | 10MR |
| 41 | 2- | 2 | ;- | ; | 2-- | 20R |
|  |  |  |  |  |  |  |

**Table S3. APR Brown Rust (*P. triticina*)**

| S.N. | Brown Rust (Seedling Response) | | | |  | APR Score |
| --- | --- | --- | --- | --- | --- | --- |
|  | **22R45 (12-5)** | **119R63 (77-1)** | **121R63-1 (77-5)** | **121R60-1 (52 or 77-9)** | **21R55**  **(104-2)** |  |
| 1 | ;1 | ;1 | 3+ | 12+ | 33+ | NG |
| 2 | ;- | 23 | 3+ | 3+ | 3+ | 20S |
| 3 | 3+ | 3+ | 3+ | 3+ | 3+ | 5R |
| 4 | 3+ | 3+ | 3+ | 3+ | 3+ | 0R |
| 5 | 3+ | 3+ | 3+ | 3+ | ;1 | - |
| 6 | 3+ | 3+ | 3+ | 3+ | 3 | 20MS |
| 7 | ;- | 3+ | 3+ | 3+ | 3+ | 40S |
| 8 | ;- | 3+ | 3+ | 3+ | 3+ | 40S |
| 9 | 3+ | 3+ | 3+ | 3+ | 3+ | 0R |
| 10 | 3+ | 3+ | 3+ | 3+ | 3+ | 40S |
| 11 | ;- | ; | ;- | ;- | ;- | 0R |
| 12 | ;- | 3+ | 3+ | ;- | ;- | 10S |
| 13 | ;- | ;1 | ;12 | 3+ | 3+ | 10MR |
| 14 | ;1 | ;1 | ;1 | ;12 | 3+ | 0R |
| 15 | ; | ;1 | ;3 | 33+ | 33+ | 0R |
| 16 | ;- | ; | ;1 | ;12 | 33+ | 0R |
| 17 | ;12+ | 3+ | 3+ | 3+ | ;12 | 5R |
| 18 | ;- | ;- | 3+ | 23 | 33+ | 10R |
| 19 | ;- | 3+ | 3+ | 3+ | ;1 | 0R |
| 20 | 33+ | ;- | ;12 | 23 | 3+ | - |
| 21 | ;12 | ; | 23 | 33+ | ;1 | - |
| 22 | ; | 3+ | 3+ | 3+ | 23 | 5MS |
| 23 | ;1 | ;1 | 33+ | 3+ | 33+ | 0R |
| 24 | ;1 | ;1 | 33+ | 33+ | ;1 | 0R |
| 25 | ;1 | 3+ | 3+ | ;- | ;1 | 0R |
| 26 | ; | 3+ | ;1 | ;1 | 3+ | 5S |
| 27 | ;- | 33+ | 3+ | 3+ | ;12 | 10R |
| 28 | ;- | 3+ | 3+ | 23 | 12 | 0R |
| 29 | ;- | 33+ | 3+ | 3+ | ;1 | 0R |
| 30 | 3+ | 3+ | 3+ | 33+ | 3+ | - |
| 31 | 3+ | 33+ | 3+ | 3+ | 12+ | - |
| 32 | ;- | 3+ | 33+ | 33+ | ;- | 0R |
| 33 | ;1 | 12 | 33+ | ;1 | 2+ | 0R |
| 34 | 3+ | 33+ | 3+ | 33+ | 3+ | 5S |
| 35 | 33+ | ;- | ; | ;- | ;- | 0R |
| 36 | ;- | 3+ | 3+ | 3+ | 3+ | 20MS |
| 37 | ;- | 3+ | 3+ | 3+ | 3+ | 0R |
| 38 | ;- | 3+ | 3+ | 3+ | 3+ | 0R |
| 39 | ;1 | 3+ | 3+ | 3+ | ; | 0R |
| 40 | ;- | ;- | ;- | ;- | ;- | 0R |
| 41 | ;- | 33+ | 3+ | 3+ | 33+ | - |
|  |  |  |  |  |  |  |

**Table S4: Seedling response, *Yr* genes in 41 salt tolerant genotypes against the pathotypes of *Puccinia striiformis* (wheat stripe/yellow rust) during 2020-21 at ICAR-IIWBR, RS, Flowerdale, Shimla**

| **S. no.** | **Variety / Line** | **Pathotype** | | | | | | | | | | | | |
| --- | --- | --- | --- | --- | --- | --- | --- | --- | --- | --- | --- | --- | --- | --- |
|  |  | **T(47S103)** | **78S84** | **110S119** | **P(46S103)** | **K(47S102)** | **7 S0** | **46S119** | **111S68** | **79S68** | **238S119** | **110S 84** | ***Yr* gene/s** | **Remarks** |
|  |  |  |  |  |  |  |  |  |  |  |  |  |  |  |
| 1 | KRL 283 | 0; | 2- | 3+ | ;- | 0; | 0; | 0; | 0; | 0; | 3+ | 3 | ***Yr9+*** |  |
| 2 | KRL 210 | 3+ | 33+ | 3+ | 3+ | 3+ | ; | 0; | 3+ | 0; | 3+ | 2- | ***Yr2+*** |  |
| 3 | KRL 213 | 2- | 2 | 3+ | ; | 3 | 0; | 3+ | 3 | 3+ | 3+ | 33+ | ***Yr2+*** |  |
| 4 | KRL 19 | 3+ | 3+ | 3+ | 3+ | 0; | 3+ | 3+ | 0; | 0; | 3+ | 0; | ***Yr2+*** |  |
| 5 | KRL 1-4 | 3+ | 3+ | 3+ | ; | 3 | ; | 0; | 3+ | 0; | 3+ | 0; | ***Yr2+*** |  |
| 6 | KRL 99 | 3+ | 3+ | 3+ | 33+ | 0; | 3+ | 3 | + | 3+ | 3+ | 3+ | ***-*** |  |
| 7 | KRL 3-4 | 3+ | 3+ | 3+ | 3+ | 0; | 3+ | 3+ | 0; | 3+ | 3+ | 3+ | ***-*** |  |
| 8 | KRL 119 | 3+ | 3 | 3+ | 3 | 3+ | 3 | 2- | 2- | 3+ | 33+ | 3+ | ***-*** |  |
| 9 | KRL 238 | 3+ | 3+ | 0; | 3+ | 0; | 3 | 3+ | 0; | ; | 0; | 0; | ***-*** |  |
| 10 | Kharchia 65 | 3+ | 3+ | 3+ | 33+ | 3+ | 3+ | 3+ | 3+ | 3+ | 3+ | 3+ | ***-*** |  |
| 11 | HD 2851 | 0; | 3+ | 3+ | ; | 3+ | 0; | 3 | 0; | 3+ | 3+ | 3+ | ***-*** |  |
| 12 | KRL 2001 | 3+ | 3+ | 3+ | 3 | 3 | 3+ | 3+ | 0; | 3+ | 3+ | 3+ | ***-*** |  |
| 13 | KRL 2002 | 3+ | 3+ | 3+ | 3+ | 0; | 0; | 3+ | 0; | 0; | 3+ | 0; | ***Yr2+*** |  |
| 14 | KRL 2003 | 3- | 3+ | 3 | 3; | 3+ | 3+ | 3 | 0; | ;0 | 3+ | 3+ | ***-*** |  |
| 15 | KRL 2004 | 0; | 3+ | 3+ | 3+ | 0; | 2- | 0; | 0; | 0; | 3+ | 0; | ***Yr2+*** |  |
| 16 | KRL 2005 | 3+ | 3- | 3 | 3N | 3 | 2- | 3+ | 0; | 33+ | 3+ | 0; | ***Yr2+*** |  |
| 17 | KRL 2006 | 3+ | 3+ | 2- | 3+ | 0; | 3 | 3+ | 3+ | 33+ | 3+ | 3+ | ***-*** |  |
| 18 | KRL 2007 | 3+ | 33+ | 3 | 3+ | 0; | 0; | 3+ | 3+; | 3+ | 3+ | 3+ | ***Yr2+*** |  |
| 19 | KRL 2008 | 3+ | 1N | 3+ | 3+ | 3+ | 0; | 3+ | 3+ | 3+ | 3+ | 0; | ***Yr2+*** |  |
| 20 | KRL 2009 | 3+ | 3 | 3+ | 3 | 0; | 0; | 33+ | 0; | 2 | 3+ | 0; | ***Yr2+*** |  |
| 21 | KRL 2010 | 0; | 3 | 3+ | 3+ | 3+ | 0; | 3+ | 3+ | 33+ | 3+ | 2- | ***Yr2+*** |  |
| 22 | KRL 2011 | 0; | 0; | 3+ | ;3- | 3+ | 0; | 0; | 2- | 3+ | 3+ | 0; | ***Yr2+*** |  |
| 23 | KRL 2012 | 3 | 1 | 3+ | 3+ | 3+ | 0; | 3+ | 3 | 0; | 3+ | 0; | ***Yr2+*** |  |
| 24 | KRL 2013 | 0; | 0; | 0; | 0; | 0; | 0; | ;- | 0; | 0; | 0; | 2- | ***Yr9+*** |  |
| 25 | KRL 2014 | 0; | 3- | 3+ | 2N | 3 | 0; | 0; | 0; | 3+ | 3+ | 33+ | ***Yr2+*** |  |
| 26 | KRL 2015 | 0; | 0; | 3+ | ;N | 0; | 0; | 0; | 0; | ;- | 3+ | ;1 | ***Yr9+*** |  |
| 27 | KRL 2016 | 3+ | 3 | 3+ | 3N | 3+ | 0; | 0; | 3+ | 3+ | 3+ | 33+ | ***Yr2+*** |  |
| 28 | KRL 2017 | 0; | 2 | 0; | 0; | 0; | 0; | 0; | 0; | 0; | 3+ | 3+ | ***Yr9+*** |  |
| 29 | KRL 2018 | 3+ | 0; | 3+ | 3+ | 0; | ; | 3+ | 0; | 0; | 3+ | ; | ***YrA+*** |  |
| 30 | KRL 2019 | 3+ | 3- | 3+ | 3+ | 0; | 0; | 3+ | 33+ | ;- | 3+ | 3+ | ***Yr2+*** |  |
| 31 | KRL 2020 | 3- | 2 | 3 | ;- | 2 | 0; | ;- | 0; | 3+ | 3+ | 0; | ***Yr2+*** |  |
| 32 | KRL 2021 | 0; | 0; | 3 | 0; | 3+ | 0; | 0; | 3 | 23 | 3+ | 2 | ***Yr2+*** |  |
| 33 | KRL 2022 | 3 | 3- | 3+ | 0; | 0; | 0; | 3 | 3+ | 2+ | 3+ | 33+ | ***Yr2+*** |  |
| 34 | KRL 2023 | 3 | 3+ | 0; | 3 | 0; | 3- | 3 | 3+ | 33+ | 3+ | 3+ | ***-*** |  |
| 35 | KRL 2024 | 0; | 0; | 3+ | ; | 0; | 0; | 0; | 0; | ;- | 3+ | 0; | ***Yr9, Yr A+*** |  |
| 36 | KRL 2025 | 0; | 3- | 3+ | 3+ | 3+ | 0; | 3+ | 3+ | 3+ | 3+ | 3+ | ***Yr2+*** |  |
| 37 | KRL 2026 | 3+ | 3+ | 3+ | 3+ | 3+ | ;- | 3+ | 3 | 3+ | 3+ | 3+ | ***Yr2+*** |  |
| 38 | KRL 2027 | 3+ | 3+ | 3+ | 3+ | 3+ | 2 | 3+ | 3+ | 3+ | 3+ | 3+ | ***-*** |  |
| 39 | KRL 2028 | 0; | 2- | 2- | ;- | 0; | 0; | 0; | 0; | 3+ | 3+ | 3+ | ***Yr2+*** |  |
| 40 | KRL 2029 | 0; | 3+ | 3+ | ;- | 0; | 0; | 0; | 0; | 0; | 3+ | 3+ | ***Yr9+*** |  |
| 41 | KRL 2030 | 3 | ;- | 3+ | 3 | 3 | 0; | 0; | ;- | 0; | 3+ | 0; | ***YrA+*** |  |

**Table S5: Seedling response, *Lr* genes in forty one salt tolerant genotypes against the pathotypes of *Puccinia triticina* (wheat leaf/brown rust) during 2020-21 at ICAR-IIWBR, RS, Flowerdale, Shimla**

| **S. no.** | **Variety/Line** | **Pathotype** | | | | | | | | | | | | | | | | | | |
| --- | --- | --- | --- | --- | --- | --- | --- | --- | --- | --- | --- | --- | --- | --- | --- | --- | --- | --- | --- | --- |
|  |  | **11** | **12A** | **12-3** | **12-5** | **12-7** | **77** | **77-1** | **77-2** | **77-5** | **77-7** | **77-8** | **77-9** | **77-10** | **104-2** | **107-1** | **108-1** | **162-1** | ***Lr* gene/s** | **Remarks** |
| 1 | KRL 283 | ;- | ;- | ; | ;1 | 3+ | ;- | ;1 | 0; | 3+ | ;12 | 0; | 12+ | ;1 | 33+ | ;- | 0; | ;12 | ***Lr26+23+10*** | 1 |
| 2 | KRL 210 | ;- | 12+ | ;1 | ;- | 3+ | ; | 23 | 3+ | 3+ | 33+ | ;- | 3+ | 3+ | 3+ | ;- | ;- | 33+ | ***Lr23+10*** | 2 |
| 3 | KRL 213 | ;- | 33+ | 3+ | 3+ | 3+ | 3+ | 3+ | ;3 | 3+ | 33+ | ;- | 3+ | 3+ | 3+ | ;- | ;- | 3 | ***Lr13+*** | 3 |
| 4 | KRL 19 | ;- | 3+ | ;1 | 3+ | 3+ | 3+ | 3+ | 2+3 | 3+ | 33+ | ;- | 3+ | 3+ | 3+ | ;- | ;- | 3 | ***Lr13+*** | 4 |
| 5 | KRL 1-4 | ;- | ;- | ;- | 3+ | 0; | 3+ | 3+ | 3+ | 3+ | 33+ | 3+ | 3+ | 3+ | ;1 | ;- | ;- | ;- | ***Lr13+*** | 5 |
| 6 | KRL 99 | 0; | 33+ | 33+ | 3+ | 3+ | 3+ | 3+ | 3+ | 3+ | 33+ | 3+ | 3+ | 3+ | 3 | 3+ | ;- | 3+ | ***Lr13+*** | 6 |
| 7 | KRL 3-4 | ;- | ;- | ;- | ;- | ;- | 3+ | 3+ | 3+ | 3+ | 3+ | 3+ | 3+ | 3+ | 3+ | ;- | ;- | 0; | ***Lr13+10+1+*** | 7 |
| 8 | KRL 119 | ;- | ;- | ;- | ;- | ;- | 3+ | 3+ | 3+ | 3+ | 33+ | 3+ | 3+ | 3+ | 3+ | ;- | ;1 | 0; | ***Lr13+10+1+*** | 8 |
| 9 | KRL 238 | ;- | 3+ | 23 | 3+ | 3+ | 3+ | 3+ | 3+ | 3+ | 33+ | ;- | 3+ | 3+ | 3+ | ;- | ; | 33+ | ***Lr13+*** | 9 |
| 10 | Kharchia 65 | 3+ | 3+ | 3+ | 3+ | 3+ | 3+ | 3+ | 3+ | 3+ | 3+ | 3+ | 3+ | 3+ | 3+ | 3+ | 33+ | 3+ | - | 10 |
| 11 | HD 2851 | ;- | ;- | ;- | ;- | ;- | ;- | ; | ;- | ;- | ;- | 0; | ;- | ;- | ;- | ;- | ;- | ;- | ***Lr24+R*** | 11 |
| 12 | KRL 2001 | ;- | ;- | X+ | ;- | ;- | 3+ | 3+ | 3+ | 3+ | 33+ | 3+ | ;- | ;- | ;- | 3+ | ;1 | 3+ | ***Lr13+2a*** | 12 |
| 13 | KRL 2002 | ;- | 33+ | ; | ;- | 3+ | ;- | ;1 | 3+ | ;12 | 3+ | ;- | 3+ | 12+ | 3+ | ;- | ;- | ;1 | ***Lr23+10*** | 13 |
| 14 | KRL 2003 | ; | ;12 | ;- | ;1 | 3+ | ;- | ;1 | 33+ | ;1 | 33+ | 0; | ;12 | ;12++ | 3+ | ;- | ; | ;1 | ***Lr23+10+*** | 14 |
| 15 | KRL 2004 | ; | ;12+ | ; | ; | ;1 | ;- | ;1 | 33+ | ;3 | 33+ | 0; | 33+ | ;12+ | 33+ | ;- | ;- | ; | ***Lr23+10+*** | 15 |
| 16 | KRL 2005 | ;- | ;1 | ;- | ;- | 33+ | ;- | ; | 23 | ;1 | ;1 | 0; | ;12 | ;12 | 33+ | ;- | ;- | ; | ***Lr23+10+*** | 16 |
| 17 | KRL 2006 | ;- | ; | ; | ;12+ | ;1 | 3+ | 3+ | 33+ | 3+ | 3+ | ; | 3+ | 3+ | ;12 | 33+ | 23 | ; | ***Lr13+3+*** | 17 |
| 18 | KRL 2007 | ; | ;12 | ;- | ;- | 3+ | ;- | ;- | 33+ | 3+ | 12+ | ;- | 23 | 12+ | 33+ | ;- | ;- | ;- | ***Lr23+10+*** | 18 |
| 19 | KRL 2008 | ;- | ; | ;1 | ;- | ;- | ;- | 3+ | 3+ | 3+ | 3+ | 3+ | 3+ | 3+ | ;1 | ;- | ; | ; | ***Lr10+1+*** | 19 |
| 20 | KRL 2009 | 0; | 3+ | ;1 | 33+ | 3+ | ;- | ;- | 3+ | ;12 | 33+ | ;- | 23 | 3+ | 3+ | ;- | ;- | ; | ***Lr23+*** | 20 |
| 21 | KRL 2010 | 0; | ;1 | ; | ;12 | ;3+ | ;1 | ; | 0; | 23 | 33+ | ;- | 33+ | 3+ | ;1 | ;- | 0; | ; | ***Lr23+3+*** | 21 |
| 22 | KRL 2011 | 0; | ;12+ | ;1 | ; | ;- | 33+ | 3+ | 0; | 3+ | 33+ | 3+ | 3+ | 3+ | 23 | ;- | ; | ;1 | ***Lr13+*** | 22 |
| 23 | KRL 2012 | ; | ; | ;12+ | ;1 | 0; | ;3+ | ;1 | ;1 | 33+ | 3+ | ; | 3+ | 3+ | 33+ | 33+ | 2+3 | ;- | ***Lr13+*** | 23 |
| 24 | KRL 2013 | ; | ;- | ; | ;1 | ;- | ;- | ;1 | ; | 33+ | ;12 | ;- | 33+ | 33+ | ;1 | 23 | ;1 | ;- | ***Lr26+23+1+*** | 24 |
| 25 | KRL 2014 | 0; | ;- | ;- | ;1 | 0; | 3+ | 3+ | 3+ | 3+ | ;- | 33+ | ;- | ;- | ;1 | 33+ | ;12 | 0; | ***Lr13+2a+1+*** | 25 |
| 26 | KRL 2015 | ;- | ;- | ; | ; | ;- | ;- | 3+ | ;- | ;1 | 3+ | 0; | ;1 | ;1 | 3+ | ; | ; | ;- | ***Lr26+1+*** | 26 |
| 27 | KRL 2016 | ;1 | ;12 | 2+3 | ;- | 33+ | 33+ | 33+ | 33+ | 3+ | 23 | 33+ | 3+ | 3+ | ;12 | ;1 | ;- | 33+ | ***Lr13+*** | 27 |
| 28 | KRL 2017 | ;- | ;- | ;- | ;- | ;- | ;- | 3+ | ; | 3+ | ;1 | 0; | 23 | ;- | 12 | ;1 | 0; | ;- | ***Lr26+1+*** | 28 |
| 29 | KRL 2018 | ;1 | ;- | ;1 | ;- | ;1 | 3+ | 33+ | 33+ | 3+ | 33+ | 33+ | 3+ | 3+ | ;1 | ; | 0; | ;- | ***Lr13+1+*** | 29 |
| 30 | KRL 2019 | ;- | 33+ | ;1 | 3+ | 3+ | 3+ | 3+ | 23 | 3+ | 23 | ;- | 33+ | 3+ | 3+ | ;- | ; | 3+ | ***Lr13+*** | 30 |
| 31 | KRL 2020 | ;1 | 33+ | ;1 | 3+ | ;- | 3+ | 33+ | 12+ | 3+ | 33+ | 23 | 3+ | ;12 | 12+ | ;1 | 2+3 | ;3 | ***Lr13+*** | 31 |
| 32 | KRL 2021 | ;- | ;1 | ;- | ;- | ;1 | ;- | 3+ | 23 | 33+ | 33+ | ; | 33+ | ;- | ;- | ;- | ;- | ;1 | ***Lr13+10+1*** | 32 |
| 33 | KRL 2022 | ;- | ;1 | ; | ;1 | 33+ | ;- | 12 | 12+ | 33+ | 2+3 | 12+ | ;1 | ; | 2+ | ;- | ;- | ;1 | ***Lr10+3+13+*** | 33 |
| 34 | KRL 2023 | ;- | 3+ | 23 | 3+ | 3+ | 3+ | 33+ | 23 | 3+ | 33+ | ;- | 33+ | ; | 3+ | ;- | 0; | 33+ | ***Lr13+3+*** | 34 |
| 35 | KRL 2024 | ;- | ;- | ;- | 33+ | 3+ | 0; | ;- | 0; | ; | ;- | ;- | ;- | 33+ | ;- | ; | ;- | ;- | ***Lr 26+*** | 35 |
| 36 | KRL 2025 | ;- | ;- | ;12 | ;- | 0; | 12 | 3+ | 33+ | 3+ | 3+ | ;12+ | 3+ | ;- | 3+ | ;- | ;- | 3+ | ***Lr13+10+*** | 36 |
| 37 | KRL 2026 | ; | ;- | ;- | ;- | 0; | 33+ | 3+ | 33+ | 3+ | 3+ | 2+3 | 3+ | 3+ | 3+ | ;- | ;- | ;- | ***Lr13+1+*** | 37 |
| 38 | KRL 2027 | ;- | ;- | ; | ;- | 0; | 23 | 3+ | 33+ | 3+ | 3+ | 23 | 3+ | 3+ | 3+ | ;- | ;- | ;- | ***Lr13+1+*** | 38 |
| 39 | KRL 2028 | ;- | ;- | ;- | ;1 | ;1 | 3+ | 3+ | 33+ | 3+ | 33+ | 33+ | 3+ | ;1 | ; | ;- | ;- | ;1 | ***Lr13+10+1+*** | 39 |
| 40 | KRL 2029 | ;- | ;- | ;- | ;- | ;- | ;- | ;- | ;- | ;- | ;- | ;- | ;- | ;- | ;- | ;- | ;- | ;- | ***Lr26+R+ Lr24+*** | 40 |
| 41 | KRL 2030 | ;- | 12+ | ;1 | ;- | 23 | 0; | 33+ | 3+ | 3+ | 3+ | 12 | 3+ | ;- | 33+ | ;- | ; | ;12 | ***Lr13+10+*** | 41 |

**Table S6:** **Seedling response, *Sr* genes in 41 salt tolerant genotypes against the pathotypes of *Puccinia graminis tritici* (wheat stem/black rust) at ICAR-IIWBR, RS, Flowerdale, Shimla.**

| **S. No.** | **Variety/Line** | **Pathotypes** | | | | | | | | | | | | | |
| --- | --- | --- | --- | --- | --- | --- | --- | --- | --- | --- | --- | --- | --- | --- | --- |
|  |  | **11** | **21A-2** | **34-1** | **40** | **40A** | **40-2** | **40-3** | **42B** | **117A-1** | **117-4** | **117-6** | **122** | **184-1** | ***Sr* genes/resistance** |
| **1** | KRL 283 | 2-- | ; | 0; | 0; | 2-- | 0; | 2-- | 0; | 0; | 0; | 0; | 0; | 0; | ***Sr31+*** |
| **2** | KRL 210 | 3+ | 22+ | 12 | 33+ | 3 | 3+ | 3+ | 3 | 12 | 12 | 22+ | 2-- | 3 | ***Sr28+*** |
| **3** | KRL 213 | 33+ | 12 | 0; | 0; | 23 | 2-- | 22+ | 2-- | 0; | 2- | 2-- | 2- | 2- | ***Sr28+*** |
| **4** | KRL 19 | 2-- | 2- | 0; | 0; | 2- | 2-- | 12 | 2-- | 0; | 0; | 0; | 0; | 0; | ***R*** |
| **5** | KRL 1-4 | 2-- | 2-- | 0; | 3+ | 0; | 0; | 22+ | 2 | 0; | 12 | 2-- | 2- | 12 | ***Sr28+*** |
| **6** | KRL 99 | 3 | ;1 | 2-- | ; | 22+ | 2-- | 2+ | 2C | 2- | 2- | 12 | 12 | - | ***Sr28+*** |
| **7** | KRL 3-4 | 3+ | 2-- | 2-- | ;1 | 3+ | 2-- | 3+ | 12C | 2-- | 23 | 2-- | 33+ | 3 | ***Sr11+*** |
| **8** | KRL 119 | 33+ | ;- | 0; | ; | 2- | ;- | 22+ | 2-- | 0; | 2-- | 2-- | 3+ | 2 | ***Sr11+*** |
| **9** | KRL 238 | 0; | 0; | 0; | ; | 22+ | 2-- | 23- | 0; | 0; | 0; | 0; | 0; | 0; | ***Sr5+11+*** |
| **10** | Kharchia 65 | 3+ | 2-- | 2 | ;- | 3+ | 33+ | 3+ | 33+ | 0; | 33+ | 2-- | 3+ | 3+ | ***-*** |
| **11** | HD 2851 | 2+ | 2-- | ; | ; | 2- | ; | 2- | 2-- | 2-- | 0; | 2-- | ; 1N | 0; | ***Sr24+*** |
| **12** | KRL 2001 | 33+ | 0; | 2-- | 2-- | 12 | 22+ | 12 | ; | 0; | 2-- | 0; | 2+C | ; | ***Sr28+*** |
| **13** | KRL 2002 | 1P2- | 2-- | 0; - | ;- | 2- | ;1 | 23 | 2-- | 2-- | 12 | 2-- | 2-- | 2-- | ***Sr30+*** |
| **14** | KRL 2003 | 33+ | 12 | 2-- | 3+ | 2- | 3+ | 3+ | 12 | 2-- | 2-- | 2-- | 22-C | 2-- | ***Sr28+*** |
| **15** | KRL 2004 | 2- | 2-- | ; 1N | ; | 2- | 2- | 2- | 2-- | 0; | 12 | 2-- | 2-C | - | ***R*** |
| **16** | KRL 2005 | 2- | 2-- | 2-- | 1P; | 2- | 2- | 2- | 12 | 2-- | 2-- | 12 | 12 | - | ***R*** |
| **17** | KRL 2006 | 1P2- 1P2+ | ; | 0; | ; | 23C | 2- | 2+C | ; | 0; | 0; | 0; | 22+ | 0; | ***Sr28*** |
| **18** | KRL 2007 | 12 | 2-- | 0; | 3+ | 2- | ; 1N | 22+C | 2- | 0; | 2-- | 12 | 2- | 2-- | ***-*** |
| **19** | KRL 2008 | 2+C | 2-- | 0; | 2- | 2- | ; | 2+C | 2 | 2- | 2- | 2-- | 2- | 0; | ***Sr11+*** |
| **20** | KRL 2009 | 33+ | 12 | ; 1N | 2- | 2- | 33+ | 2- | ; | 2- | 0; | 0; | 2- | 0; | ***Sr28+*** |
| **21** | KRL 2010 | 33+ | ; 1N | 2-- | 3 | 2CN | 3+C | 3+ | ; | 0; | 0; | 12 | 2-- | 0; | ***Sr28+*** |
| **22** | KRL 2011 | 2C | ; | 0; | 0; | 2- | 0; | 2+C | 0; | 0; | 0; | 0; | ; | 0; | ***Sr11+*** |
| **23** | KRL 2012 | 12 | ; | 0; | 0; | 12 | 0; | 2+C | 0; | 0; | 1P0; | 0; | ;- | 2-- | ***-*** |
| **24** | KRL 2013 | 2-- | ; | 0; | 0; | 2- | 0; | 2C | 0; | 0; | 0; | ;1 | ;1 | 0; | ***Sr31+*** |
| **25** | KRL 2014 | 2-- | 0; | 0; | 0; | 2-- | 0; | 12C | 0; | 0; | 0; | ; | ; | 0; | ***Sr30+*** |
| **26** | KRL 2015 | 12 | 0; | 0; | 0; | 2- | ;- | 2-- | 2- | 2-- | 0; | 2-- | 0; | 0; | ***Sr31+*** |
| **27** | KRL 2016 | 0; | ; | 0; | 0; | 2- | 0; | 3+ | 12C | 0; | 2-- | 0; | ;1 | ;1 | ***Sr11+*** |
| **28** | KRL 2017 | 0; | 2-- | 0; | 2- | 2-- | ;1 | 2- | 0; | 2-- | 0; | 0; | 2- | 0; | ***Sr31+*** |
| **29** | KRL 2018 | 0; | ; | 0; | 0; | 0; | 0; | 2+C | 0; | 0; | 0; | 0; | ; | 0; | ***Sr57+11+*** |
| **30** | KRL 2019 | 2-- | 2-- | 0; | 2-- | 2- | 2- | 12C | 2-- | 0; | 2-- | ;- | ;1 | 2- | ***Sr11+*** |
| **31** | KRL 2020 | 12 | ; | 0; | ; | ;1 | 0; | 33+ | 0; | 2- | 22+ | ;1N | 3+C | 12 | ***Sr7b+11+*** |
| **32** | KRL 2021 | 2- | 0; | 0; | ; | 2+ | 0; | 2C | 0; | 0; | 0; | 0; | 2- | 0; | ***-*** |
| **33** | KRL 2022 | 3+ | 0; | 0; | 3+ | 2- | 33+ | 3+ | 0; | 2-- | 0; | 0; | 22+ | 0; | ***Sr28+*** |
| **34** | KRL 2023 | 0; | 2 | 0; | ; | 0; | ;- | 12 | 0; | 0; | 0; | ;- | 0; | 0; | ***-*** |
| **35** | KRL 2024 | 2 | 2 | 0; | ; | 2-- | ;- | 2- | 2-- | 2-- | 2-- | 2-- | 2-- | 0; | ***Sr31+*** |
| **36** | KRL 2025 | 2 | 2 | 0; | ; | 2-- | ;- | 12 | ;- | 0; | 0; | ;- | 2-- | 0; | ***-*** |
| **37** | KRL 2026 | 2+C | 2 | 0; | ; | 33+ | 2-- | 2+C | 2+C | 2-- | 2 | 2-- | 3+ | 2- | ***Sr7b+11+*** |
| **38** | KRL 2027 | 22+C | 2 | 0; | ; | 3+C | 2- | 3 | 2C | 2-- | 2-- | 12 | 3+ | 3 | ***Sr7b+11+*** |
| **39** | KRL 2028 | 12C | ; | 0; | 0; | 3+ | 0; | 2+C | ;N | 0; | ; | ;1 | 0; | 3+C | ***Sr7b+11+*** |
| **40** | KRL 2029 | 0; | ; | 0; | ;- | 2- | 0; | 2- | 2-- | ; | ;- | ;- | 2-- | 2- | ***Sr 31+24+*** |
| **41** | KRL 2030 | 2- | ; | 0; | ; | 2 | 0; | 2- | 2-- | 0; | ;- | ;- | 2-- | 2-- | ***-*** |
